# Effectiveness of a protein-supplemented very-low-calorie diet program for weight loss: a randomized controlled trial in South Korea

**DOI:** 10.3389/fnut.2024.1370737

**Published:** 2024-09-12

**Authors:** Eunbyul Cho, Sohye Kim, Hwa Jung Kim, Belong Cho, Jin Ho Park, Hyuktae Kwon, Ju Young Kim, Yumi Go, Dong Gyun Kang, Eunyoung Shin, Sumi Lee, Siye Gil, Hyerim Kim, Jihyun Ahn, Joo Young Kim, WonJoo Jung, Eunyoung Go

**Affiliations:** ^1^Department of Family Medicine, Seoul National University Bundang Hospital, Seongnam-si, Republic of Korea; ^2^Department of Clinical Epidemiology and Biostatistics, Asan Medical Center, University of Ulsan College of Medicine, Seoul, Republic of Korea; ^3^Department of Family Medicine, Seoul National University Hospital, Seoul National University College of Medicine, Seoul, Republic of Korea; ^4^Bionutrion, Seoul, Republic of Korea; ^5^Public Health Care Headquarters, Seoul National University Bundang Hospital, Seongnam-si, Republic of Korea; ^6^Uahn Clinic, Seoul, Republic of Korea; ^7^LohasMedi, Asan-si, Republic of Korea

**Keywords:** high-protein diet, very-low-calorie diet, meal replacement, obesity, waist circumference

## Abstract

**Introduction:**

Weight-loss strategies through meal replacements are effective and sustainable options. However, few studies have assessed their effects on weight loss including body composition through protein-supplemented meal replacements targeting the Asian population, including Koreans. This study aimed to assess the effectiveness and safety of a protein-supplemented very-low-calorie diet (PSVLCD) for weight reduction and changes in body composition in individuals with obesity over a 12-month long-term period.

**Methods:**

In total, 106 participants with obesity were randomly assigned to a PSVLCD or control group (food-based calorie-restricted diet). Body weight, waist circumference, body composition, and blood marker levels were measured throughout the study. Statistical analyses were performed to compare outcomes between the groups.

**Results:**

Among the 106 participants, 84 completed the 12-month follow-up. Intention-to-treat analysis showed that the mean weight loss from baseline to 12 months was −6.86 kg (8.21% of baseline weight) in the PSVLCD group and − 4.66 kg (5.47% of initial body weight) in the control group; the difference was −2.20 kg with a marginally significant interval (95% confidence interval [CI], −4.90; 0.50). Waist circumference (−8.35 cm vs. -4.85 cm; mean difference, −3.49 cm; 95% CI, −6.48 to −0.50) and visceral fat area (−28.28 cm^2^ vs. −13.26 cm^2^; mean difference, −15.03cm^2^; 95% CI, −29.01 to −1.04) also significantly decreased in the PSVLCD group at 12 months.

**Discussion:**

The PSVLCD group demonstrated a substantial initial reduction in waist circumference that was sustained over the study period, alongside a marginally significant decrease in weight. These findings suggest that a protein-supplemented very-low-calorie diet may be an effective strategy for long-term weight management and body composition improvement in individuals with obesity.

**Clinical trial registration:**

ClinicalTrials.gov, identififer NCT04597788.

## 1 Introduction

Obesity, defined as abnormal fat accumulation and classified as a chronic disease, has been a major global health challenge (1). It is a significant risk factor for diabetes, cardiovascular diseases, and several types of cancers (2). In South Korea, the prevalence of obesity, defined as a body mass index (BMI) of >25 kg/m^2^ (3), has steadily increased from 29.7% in 2009 to 32.5% in 2018. Remarkably, a sharp increase in obesity, especially class 2 obesity, defined as a BMI of >35 kg/m^2^, has been observed among children, adolescents, and young adults (2). According to the Korean Society for the Study of Obesity guidelines, lifestyle interventions, including a low-calorie diet with moderate physical activities, are the cornerstone for its management (2). However, these interventions cannot be easily implemented in many clinics or hospitals because of time constraints and insurance coverage issues. The healthcare insurance system in South Korea does not adjust medical fees based on the complexity or duration of medical services provided. Consequently, healthcare providers do not receive additional compensation for spending extra time with patients.

Meal replacements can help manage obesity as they help in significantly reducing body weight, BMI, body fat, fasting glucose, glycated hemoglobin (HbA1c), and systolic and diastolic blood pressures (BPs) in patients with type 2 diabetes and obesity (4). In a meta-analysis, Astbury et al. reported that meal replacement resulted in a significant difference of −1.44 kg compared with other diets. When meal replacement was used with support such as dietary advice and other behavioral strategies, the difference was −2.22 kg compared with other diets with support and − 3.87 kg compared with other unsupported diets. In addition, when high-level support, such as customized counseling for patients, was added at 2-week intervals, the difference between diet with meal replacements and regular support was −6.13 kg, and the effect was maintained for up to 1 year; this suggested that meal replacement with support is a useful method for weight loss and maintenance (5).

Very-low-calorie diets (VLCDs, <900 kcal/day) (6) using total diet replacements such as soups and shakes have proven beneficial for weight loss and diabetes remission for at least 2 years among people with obesity and type 2 diabetes (7, 8). Previous research has proven the possibility of successful primary care-led weight management programs and long-term remission of diabetes achieved by non-pharmacological, nonsurgical treatments. However, there have also been concerns regarding the adverse effects of VLCD, including a decrease in skeletal muscle mass (9). Some successful strategies for ameliorating this concern are protein supplementation and physical exercise (10). Several meta-analyses have assessed the effects of high-protein diets on weight and body fat loss. In a short-term study of <1 year, high-protein diets containing 16–45% of protein for daily intake were associated with greater weight loss, reduced fat mass, and/or lean mass preservation compared with normal diets containing 5–23% protein (11, 12). In a long-term study of >1 year, a high-protein diet resulted in greater weight loss and body fat loss than a normal protein diet (13, 14). A high-protein diet can also be a successful strategy to help prevent and treat obesity because it increases satiety and reduces weight and fat mass compared with standard protein diets (15). Although this difference seemed to be attenuated after 1 year, partly owing to decreased dietary adherence, several studies have suggested that a higher increase in dietary protein intake was associated with greater long-term weight loss at 2 years (16).

Weight-loss strategies through protein-supplemented meal replacements are effective and sustainable options; however, there have been limited studies conducted on populations in East Asia, including among Koreans who have different obesity standards compared with Western populations, to investigate the effects on weight loss and long-term weight management. Most studies using meal replacements were conducted over a relatively short-term period of 12 weeks (17–19); one study with a 1-year duration showed average weight loss of −1.75 kg in intervention group through improvements in glucose levels of the participants (20). Thus, this study aimed to evaluate the effectiveness and safety of protein-supplemented VLCD with a stepped food reintroduction program on body weight reduction and body composition changes among people with obesity in Korea.

## 2 Materials and methods

### 2.1 Participants and study materials

This study was conducted between December 2020 and October 2022 at two tertiary hospitals in South Korea. The reporting in this study aligned with the Consolidated Standards of Reporting Trials reporting guidelines.

Eligible trial participants were randomly assigned to an intervention group (consisting of a 4-month protein-supplemented VLCD followed by an 8-month low-calorie diet with 1 week per month of intermittent VLCD using meal replacements) or a control group (consisting of daily food-based calorie restriction diet for 12 months). The PSVLCD was selected based on previous research demonstrating its effectiveness in rapid weight loss and maintenance of lean mass (21). The combination of a low-calorie diet with intermittent VLCD was included to assess long-term weight maintenance and improvements in metabolic health (22). Randomization stratified by sex, age group (≥65 years vs. <65 years), and BMI group (≥30 kg/m^2^ vs. <30 kg/m^2^) was generated using a random number generator by the study coordinator. For the stratification process, a BMI cutoff of 30 was used to define severe obesity. Stratifying participants by age (<65 years vs. ≥65 years) was based on the need to differentiate between younger and older adults, who may have different metabolic profiles, in accordance with the World Health Organization (WHO) definition of elderly (65 years and older).

Participants eligible for the study were men and women aged 19–70 years with obesity (BMI: 25–39.9 kg/m^2^) or who were overweight (BMI: 23–24.9 kg/m^2^) with abdominal obesity, defined by waist circumference greater than 85 cm for women and greater than 90 cm for men, according to the Asia-Pacific obesity diagnostic criteria and Korean guideline (23). Recruitment was conducted through local flyers and advertisements.

Inclusion criteria:

- Men and women aged 19–70 years
- BMI of 25–39.9 kg/m^2^ (obesity)
- BMI of 23–24.9 kg/m^2^ (overweight) with abdominal obesity (waist circumference > 85 cm for women, >90 cm for men)

Exclusion criteria:

- Weight changes of ±5% within the past 6 months
- Participation in any weight-loss program 6 months prior to screening
- Use of medications that could affect weight (weight-loss drugs: orlistat, lorcaserin, liraglutide, phentermine, topiramate, bupropion, naltrexone; steroid hormones; other dietary supplements)
- Prescriptions for medications that could cause hypoglycemia (e.g., insulin, sulfonylurea)
- Pregnant or lactating within the past 12 months or planning to become pregnant
- Diagnosis of an eating disorder, alcohol use disorder, or uncontrolled mood disorder
- Chronic renal failure (defined by an estimated glomerular filtration rate < 30 mL/min)
- Diagnosis of gallstones

### 2.2 Exercise control and physical activity assessment

Participants were encouraged to increase their physical activity to up to 30 min of moderate-intensity exercise at least five times a week and to perform two resistance exercises per week. However, physical activities were not supervised or monitored for feedback. To estimate the physical activity levels of the participants, we utilized the Global Physical Activity Questionnaire (GPAQ), developed by the WHO. The GPAQ is a validated tool designed to assess physical activity across various domains, including work, transport, and leisure, as well as sedentary behavior. The GPAQ collects data on the frequency (days per week), duration (minutes per day), and intensity of physical activities. These activities are then converted into Metabolic Equivalent of Task (MET) minutes per week to quantify the participants’ total physical activity levels. The analysis of GPAQ data was conducted following the guidelines provided in the GPAQ Analysis Guide by the WHO. The energy expenditure for each participant was estimated based on their reported physical activities, providing a comprehensive view of their overall physical activity patterns.

All participants provided written informed consent before participating in the study. All research procedures conducted in this trial were in accordance with a protocol approved by the Institutional Review Board (IRB) of Seoul National University Bundang Hospital (IRB No. B-2004-604-003) as well as by Seoul National University Hospital (IRB No. 2106–004 -1223) and adhered to the Declaration of Helsinki.

Participants who were randomized to the intervention group were provided meal replacements at every visit free of charge during the entire study period. To ensure blinding, randomization was conducted by the study coordinator using a random blind number generator. This process stratified participants by sex, age group (≥65 years vs. <65 years), and BMI group (≥30 kg/m2 vs. <30 kg/m2). Blinding was maintained throughout the study duration to prevent bias in the allocation of interventions and data collection. However, it should be noted that blinding between the intervention and control groups was not feasible in this study. The control group did not receive the protein supplement. The meal replacements comprised three sachets daily for women and four sachets daily for men for the initial total replacement, and no participants reported any allergic reactions to the sachets. One sachet of meal replacement included 20 g of protein (20 g of isolated soy protein), 2.4 g of mixed grain powder, multivitamin powder with a total of 110 kcal, and three different flavors (sweet pumpkin, caramel, and jujube). These protein-supplemented meal replacements were recommended to be consumed as mixed with 200 mL of low-fat milk, fat-free milk, or soymilk (approximately 5–6 g of protein) to give 25 g protein and 200 kcal per sachet. In addition, 25 g of dietary fiber, one tablet of multivitamins, and 1 g of omega-3 with 2000 IU of vitamin D per day were recommended to avoid essential nutrient deficiency during the weight-loss phase. Meal replacements were prepared using Bionutrion^®^ and approved as a functional health food by the Ministry of Food and Drug Safety for this research.

### 2.3 Study design and intervention

The intervention program included a 4-month weight-loss phase and an 8-month weight-loss maintenance phase. During the weight-loss period, the participants followed an initial total diet replacement with a protein-supplemented very-low-calorie diet (PSVLCD) using meal replacements for 2 weeks, followed by 8 weeks of food reintroduction with two or three meal replacements and 6 weeks of food reintroduction with one or two meal replacements. During the initial weight-loss phase, a protein intake of 1.5 g/kg of body weight per day was prescribed with meal replacements.

All participants were encouraged to submit their daily intake through food photos or lists of the types and amounts of food consumed; additionally, they were provided individual nutritional counseling by registered dietitians at least two times per week through a customized mobile application using the freely available KakaoTalk Channel^1^ for this study. The counseling focused on the importance of adhering to the structured meal-replacement plan in the initial stages. Dietitians calculated the approximate calorie and protein amounts of the foods from the photos or food lists submitted by participants. Based on this information, recommendations were provided for healthy-eating habits including suggestions for healthy, very-low-calorie meal recommendations in the food reintroduction phase. Monitoring dietary therapy online provides convenience and accessibility to both dietitians and subjects. It enables flexible scheduling, removes geographical barriers, and fosters consistent follow-up and support. In addition, the participants were encouraged to increase their physical activities to up to 30 min of moderate intensity at least five times a week and two resistance exercises per week. However, physical activities were not supervised or monitored for feedback.

When the participants entered the maintenance phase, they were recommended to adhere to a low-calorie food-based diet consisting of 1,200–1,500 kcal per day for women and 1,500–1,800 kcal per day for men. An intermittent PSVLCD using partial meal replacements for 1 week per month was adopted.

If a weight regain of >2 kg occurred, an extension of the intermittent PSVLCD was recommended for 2 weeks per month, and if a weight regain of more than 4 kg occurred, the extension was recommended for 3–4 weeks per month.

The control group was instructed to follow a food-based calorie-restricted diet of 1,500–1,800 kcal per day for men and 1,200–1,500 kcal per day for women according to the Korean Society for the Study of Obesity guidelines (2). Protein was recommended as a food-based recipe at 1 g/kg of the ideal body weight. The participants were encouraged to submit data regarding their daily food intake (by photo or writing a food journal) and weight through the online community application and were provided online education and counseling with dietitians twice per week during the total study period.

### 2.4 Study outcomes and measurements

The primary outcome was the difference in body weight from baseline to 12 months between the two groups. The secondary outcomes included changes in waist circumference, body fat mass, lean body mass, and cardiometabolic profiles.

Body composition was measured automatically using bioelectrical impedance analysis (BIA, InBody 770; InBody, Seoul, South Korea) at each visit, with the participants in a standing position with light clothing, fasting, and an empty bladder. Impedance was measured for five segments of the body, trunk, right and left arms, and right and left legs. Using the manufacturer’s algorithm, fat and muscle masses of the total body, arms and legs were calculated separately. The visceral fat area values were derived from a regression formula used to measure segmental impedance. We collected the data of skeletal muscle mass, fat mass, percent body fat and visceral fat area, which their accuracies were validated compared with CT scanning (24) and dual-energy X-ray absorptiometry (25).

Body weight was measured using a digital scale to the nearest 0.1 kg, with the participants wearing lightweight clothes. Height was measured barefoot to the nearest 0.1 cm using a stadiometer. Waist circumference was measured using a plastic measuring tape to the nearest 0.1 cm at the midline between the lowest rib margin and the anterior superior iliac spine while standing after a normal expiration. Systolic and diastolic BPs were measured in the seated position using an automated sphygmomanometer (two measurements and an average were obtained). The average systolic and diastolic values were calculated for analysis. The study outcomes were measured at 0, 0.5, 2, 4, 6, 8, 10, and 12 months.

Blood samples after 10 h of fasting were tested to measure fasting glucose, HbA1C, total cholesterol, high-density cholesterol, triglyceride (TG), and low-density cholesterol levels. The levels of aspartate and alanine aminotransferase (AST and ALT), gamma-glutamyl transferase (GGT), and uric acid and eGFR were calculated using the creatinine-based Chronic Kidney Disease Epidemiology Collaboration equation (26). The hepatic steatosis index was derived to screen for nonalcoholic fatty liver disease using the ALT/AST ratio, sex, BMI, and diabetes (27). Insulin resistance was measured using the homeostasis model assessment of insulin resistance (HOMA-IR) (28).

Dual-energy X-ray absorptiometry with Lunar Prodigy Advance (GE Lunar, Madison, WI, United States) was performed to assess the bone mineral density of the entire left hip and anterior–posterior lumbar spine (L1-L4) (29).

The questionnaires completed under the supervision of study coordinators, including the Korean version of the Obesity-related Quality of Life Scale (30), dietary pattern evaluation tool (31), Global Physical Activity Questionnaire (32), Korean version of the Hospital Anxiety and Depression Scale (33), and Korean version of the Pittsburgh Sleep Quality Index (34), were administered at baseline, 4 months, 8 months, and at the end of the study.

### 2.5 Sample size calculation

We estimated that a target sample size of 106 participants (53 per group) would provide 90% power to detect significant differences between two groups at a 2-sided level of 5% with a 30% dropout rate. The proposed group difference of 4.9 kg in body weight (±6.3) was determined from a previously published meta-analysis and total diet replacement trials for remission of diabetes (7, 8, 15). A meta-analysis showed that VLCD combined with the behavioral program achieved −3.9 kg (95% CI -6.7 to −1.1) reduction compared with the behavioral program only (15). The mean weight loss of VLCD was reported to be −10.3 kg (7–10% of initial weight loss) among people with average BMI of 35–38 kg/m^2^. In South Korea, the cutoff point of the body mass index for obesity is 25 kg/m^2^ (2), whereas the baseline BMI in the above studies was 38.2 kg/m^2^. Furthermore, the duration of VLCD, including total meal replacements, was shorter (4 months vs. 6 months or more) than that in previous studies. Considering a smaller diagnostic cutoff of obesity (BMI 25 kg/m^2^ vs. BMI 30 kg/m^2^) and relatively short duration of VLCD, including total meal replacements, we expected smaller weight loss than that reported in previous trials, which was −7.5 kg (SD 7) in the intervention group and − 2.6 kg (SD 5.4) in the control group.

A parallel two-group design was used to test whether the intervention group mean was different from the control mean (H0: μ1 - μ2 = 0 versus H1: μ1 - μ2 ≠ 0). The comparison was made using a two-sided, two-sample, unequal-variance t-test, with a Type I error rate (α) of 0.05. The standard deviation for intervention group assumed to be 7.0 and the standard deviation for control group was assumed to be 5.4. To detect a difference in means of μ1 - μ2 = −7.5 + 2.6 = −4.9 with 90% power, the number of needed subjects was 36 in each group using PASS 15 software (NCSS, Kaysville, Utah, United States).

### 2.6 Statistical analysis

An intention-to-treat (ITT) analysis was performed to compare longitudinal changes between the intervention and control groups. The ITT analysis included all participants who were originally randomized, had uploaded their food intake data at least once, and had completed the 2-week follow-up visit after randomization. To model the longitudinal data, we used linear mixed-effects models. The fixed effects included the intervention group and time points (0, 0.5, 2, 4, 6, 8, 10, and 12 months for weight, waist circumference, body fat mass, and lean body mass). Participants were included as random effects to account for individual variability and repeated measures over time. The interaction between the group and time was also included in the model to assess differential changes over time between the two groups.

When the *p*-value for the interaction term between the group and time was less than 0.05, indicating a significant difference in trajectories, *post-hoc* comparisons between the groups at each time point were conducted using Bonferroni adjustments to correct for multiple comparisons. This adjustment helped to control the family-wise error rate and provided more accurate results. Within-group changes over time were analyzed using repeated-measures linear mixed models. In these models, the intervention group and time were included as fixed effects, while time was treated as a repeated measure. This approach allowed us to account for the correlation of measurements within the same participant over different time points.

Statistical analyses were performed using SAS statistical software (version 9.4; SAS Institute, Cary, NC, United States). Statistical significance was set at a two-sided *p*-value of <0.05. By providing these additional details and clarifications, we aimed to ensure the transparency and rigor of the statistical methods used in this study.

## 3 Results

### 3.1 Participants’ characteristics

**Figure 1:**
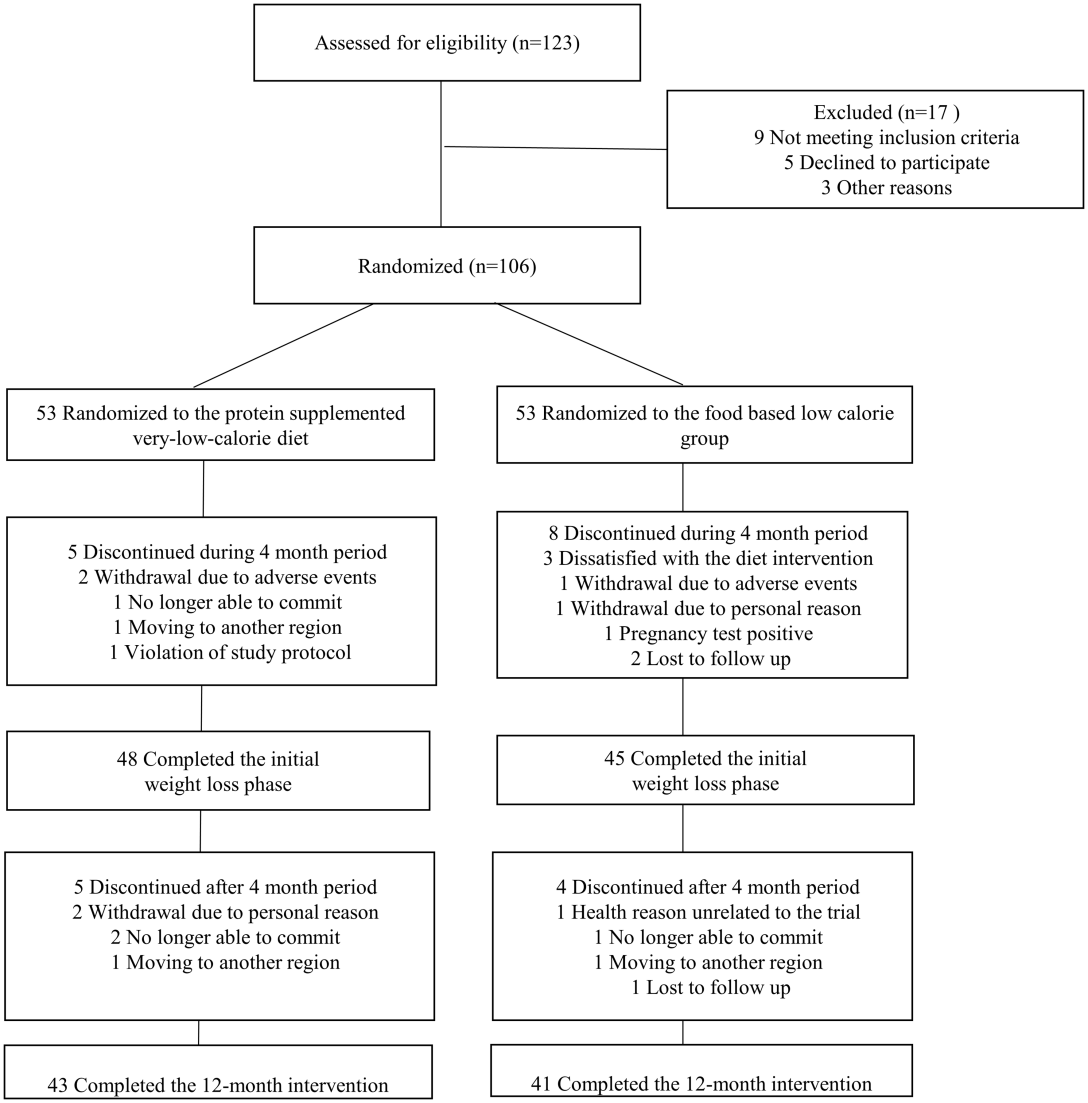
Flow of study participants.

The data on the number of participants screened, excluded, randomized, and included in the analysis are shown in Figure 1. A total of 106 participants were randomized to the PSVLCD (*n* = 53) or control group (*n* = 53), among whom 84 completed the study. The attrition rate was 17% in the PSVLCD group and < 35% in the control group (*p* = 0.045). The following results were based on an intention-to-treat analysis of the participants with baseline data.

The participants had a mean age of 43 ± 10.9 years, a mean weight of 83.4 ± 26.2 kg, a mean BMI of 30.6 ± 4.3 kg/m^2^, and a mean waist circumference of 99.7 ± 10.3 cm. The main clinical characteristics of the study population are summarized in Table 1. There were no significant differences in demographic and anthropometric measurements, cardiometabolic risk factors, and bone mineral density between the PSVLCD and control groups. The PSVLCD group showed more overeating patterns than the control group. However, there were no significant differences in other dietary patterns, mood, sleep, and obesity-related quality of life.

**Table 1:** Baseline characteristics of all participants.

Variables	PSVLCD group (*N* = 48)	Control group (*N* = 45)	*p*-value
**Demographic and anthropometric measurements**			
Female	32 (66.7)	32 (71.1)	0.644
Age, years	42.56 (10.24)	43.71 (11.59)	0.613
Body weight, kg	83.48 (15.65)	84.44 (16.92)	0.759
Body mass index, kg/m^2^	30.46 (3.98)	30.88 (4.68)	0.640
Overweight, n (%)	1 (2)	3 (7)	0.418
Grade 1 Obesity, n (%)	25 (52)	19 (42)	
Grade 2 Obesity, n (%)	22 (46)	23 (51)	
Waist circumference, cm	100.10(9.42)	99.55 (10.51)	0.959
Body fat mass, kg	31.83 (9.06)	33.02 (9.52)	0.49
Lean body mass, kg	28.70 (6.54)	29.36 (8.41)	0.898
Percent body fat, %	37.98 (6.96)	38.84 (5.51)	0.508
Visceral fat area, cm^2^	121.46 (44.02)	125.95 (43.79)	0.696
**Cardiometabolic risk factors**			
Systolic blood pressure, mmHg	130.17(17.16)	128.44 (14.33)	0.397
Diastolic blood pressure, mmHg	81.63 (12.12)	78.96 (9.10)	0.665
Glucose, mg/dL	105.31 (17.02)	107.91 (20.13)	0.502
Glycated hemoglobin, %	5.60 (0.58)	5.83 (0.64)	0.081
HOMA-IR	3.42 (3.26)	2.96 (1.29)	0.382
Total cholesterol, mg/dL	200.47 (33.75)	191.32 (35.91)	0.232
HDL cholesterol, mg/dL	53.98 (12.62)	53.53 (10.93)	0.856
LDL cholesterol, mg/dL	128.04 (27.51)	118.07 (32.28)	0.112
Triglyceride, mg/dL	122.40 (47.59)	125.69 (56.03)	0.76
AST, IU/L	28.42 (18.13)	26.51 (17.36)	0.606
ALT, IU/L	42.42 (47.23)	34.13 (31.18)	0.324
GGT, U/L	32.94 (26.61)	33.04 (32.12)	0.986
Hepatic steatosis index	42.27 (5.29)	42.14 (6.98)	0.105
eGFR, ml/min/1.73m^2^	108.39 (12.82)	107.22 (12.64)	0.659
Uric acid, mg/dL	6.29 (1.90)	5.51 (1.19)	0.021
**Bone mineral density**			
Femoral neck	0.30 (1.24)	0.41 (1.59)	0.700
Lumbar spine	0.76 (1.01)	0.72 (1.04)	0.870
**Questionnaires**			
GPAQ, median (IQR)	630 (345, 1,560)	720 (180, 1,440)	0.688
**Dietary pattern evaluation tool**			
Overeating	3.54 (0.59)	3.26 (0.70)	0.046
High fat intake	3.00 (0.76)	2.83 (0.73)	0.304
Nutritional imbalance	3.04 (0.56)	2.99 (0.75)	0.752
Impulsive eating	3.00 (0.84)	2.68 (1.01)	0.108
**HADS**			
Anxiety	5.90 (3.63)	5.62 (3.69)	0.720
Depression	7.40 (3.27)	7.40 (3.85)	0.996
PSQI	5.94 (2.84)	6.39 (3.37)	0.492
KOQOL	40.78 (9.52)	39.86 (9.95)	0.656

All such values are presented as mean ± SE. Student’s t-test for continuous variables and the chi-square test for categorical variables were used to compare the difference between the groups. PSVLCD, protein-supplemented very-low-calorie diet program; HOMA-IR: homeostasis model of assessment of insulin resistance; eGFR, estimated glomerular filtration rate; GPAQ: Global physical activity questionnaires; HADS, Hospital Anxiety and Depression Scale; PSQI, Pittsburg Sleep Quality Index; KOQOL, Korean version of Obesity-related Quality of Life Scale. HOMA-IR is calculated as the level of fasting glucose (measured in millimoles per liter) times the level of fasting insulin (measured in microunits per milliliter) divided by 22.5. Hepatic steatosis index is a screening tool reflecting nonalcoholic fatty liver disease and calculated using the following formula: 8 × (ALT/AST ratio) + BMI (+2, if female; +2, if diabetes mellitus). eGFR is calculated using the Chronic Kidney Disease Epidemiology Collaboration equation. Normal laboratory ranges are as follows: Glucose: 70–110 mg/dL; Total Cholesterol: 0–200 mg/dL; HDL Cholesterol: Men: 35–55 mg/dL, Women: 45–65 mg/dL; LDL Cholesterol: Men: 55–155 mg/dL, Women: 55–165 mg/dL; Triglycerides (TG): 0–150 mg/dL; AST: 0–40 IU/L; ALT: 0–40 IU/L; GGT: Men: 11–63 U/L, Women: 8–35 U/L; eGFR: >60 mL/min/1.73 m^2^.

### 3.2 Changes in body weight and body composition

**Figure 2:**
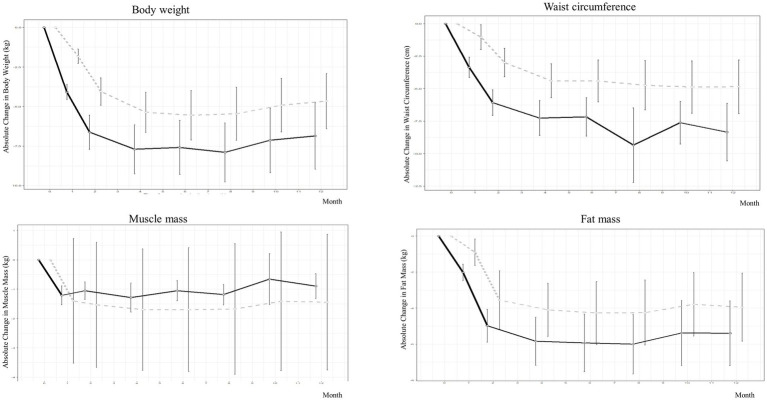
Weight and body composition changes in people with obesity during protein-supplemented very-low-calorie-diet program trial.

**Figure 3:**
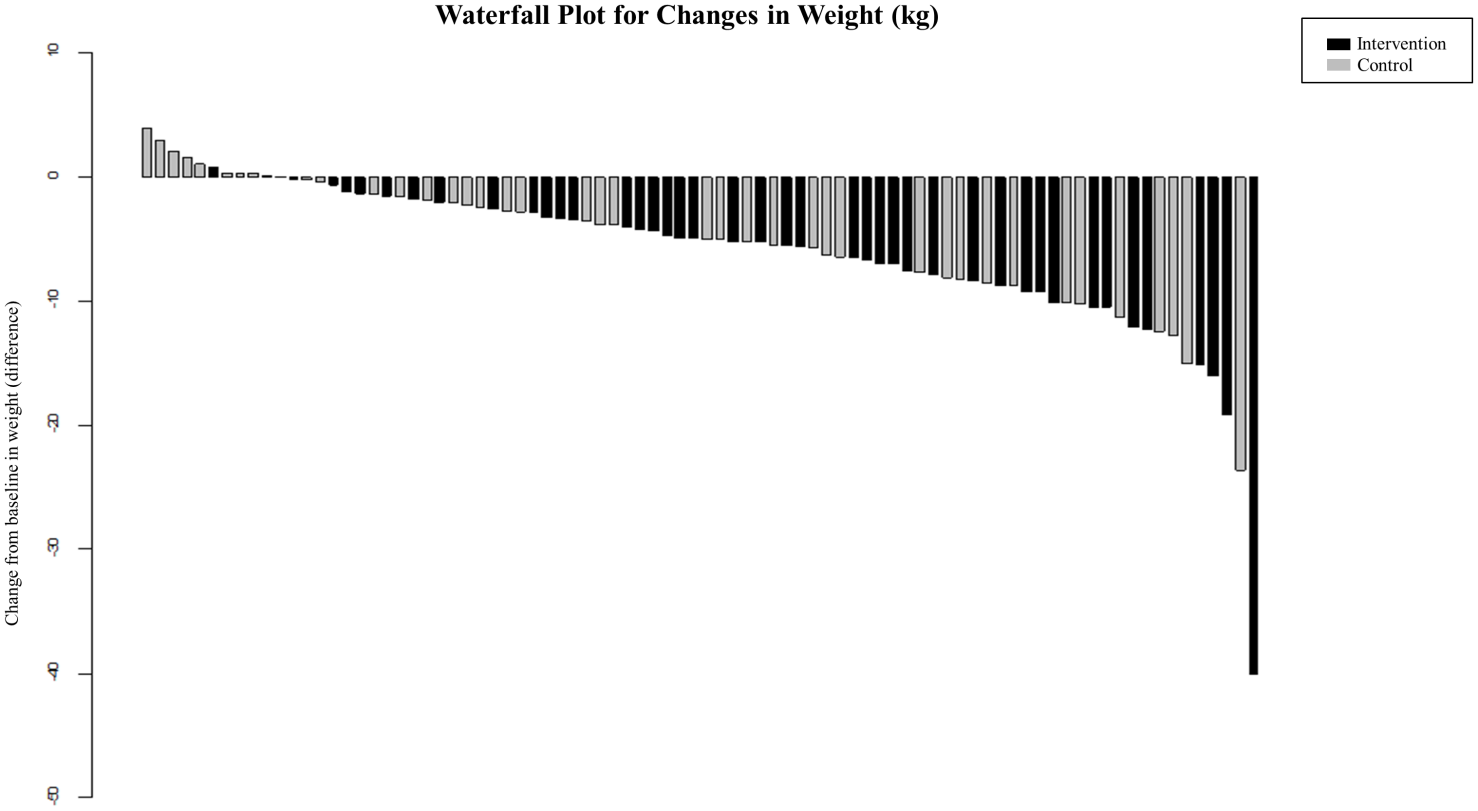
Percentage and cumulative percentage of achieving significant weight loss in people with obesity during protein-supplemented very-low-calorie-diet program trial.

As shown in Table 2, Supplementary Table S1, and Figures 2, 3, during the 4-month weight-loss phase, the PSVLCD group showed a significant body weight difference from baseline (−7.70 ± 5.33 kg vs. -5.36 ± 4.26 kg *p* = 0.022), percentage of weight loss from baseline (−9.23 ± 5.67% vs. -6.08 ± 4.45% *p* = 0.004), and waist circumference reduction (−7.27 ± 4.64 cm vs. -4.40 ± 4.38 cm *p* = 0.003) compared with the control group. On completion of the study, the participants in the PSVLCD group lost an average of −6.86 ± 6.83 kg, whereas those in the control group lost an average of −4.66 ± 5.50 kg, with a marginally significant difference between the groups (−2.20 kg; 95% CI, −4.9; 0.5). Remarkably, the PSVLCD group achieved a significantly greater reduction in waist circumference (−8.35 ± 7.21 cm vs. −4.85 ± 6.53 cm, *p* = 0.023) and visceral fat area (−28.28 ± 32.08 cm^2^ vs. −13.26 ± 23.10 cm^2^, *p* = 0.043) than the control group. Regarding cholesterol levels, the PSVLCD group showed a mean baseline total cholesterol level of 200.47 ± 33.75 mg/dL, which slightly increased to 202.56 ± 33.91 mg/dL after 12 months. Similarly, the control group had a mean baseline total cholesterol level of 191.32 ± 35.91 mg/dL, which also slightly increased to 193.54 ± 33.94 mg/dL after 12 months. These changes represent small increases in total cholesterol for both groups, and the differences between groups were not statistically significant (*p* = 0.980). Percentage of weight loss from baseline in the PSVLCD group reached its lowest point at 8 months, with a reduction of 9.47% (6.22% in control group, *p* = 0.019). However, this reduction was not maintained at the completion of study, with a reduction of 8.21% (5.47% in control group, *p* = 0.067). Visceral fat area measured by BIA reached its lowest point at 6 months (−32.28 ± 27.11 cm^2^ vs. −15.01 ± 22.99 cm^2^, *p* = 0.008), followed by slight fluctuations up to 12 months. However, the evaluation at 12 months indicated that the visceral fat area remained significantly lower compared to the control group, demonstrating sustained improvement. We performed the correlation analysis according to gender and the correlation coefficients were 0.538 for men and 0.701 for women. In general, correlation coefficient was 0.687.

**Table 2:** Body weight and body composition with the diet intervention.

Variables	PSVLCD group (*N* = 48)				Control group (*N* = 45)				Difference between groups (95% CI)	*p*-value
	Baseline	12 months	change	*p*-value	Baseline	12 months	change	*p*-value		
Body weight, kg	83.48 (15.65)	76.59 (14.72)	−6.86 (6.83)	<0.0001	84.44 (16.92)	80.01 (17.42)	−4.66 (5.50)	<0.0001	−2.20 (−4.9 to 0.5)	0.109
Percentage of weight loss (%)	100	91.79 (7.11)	−8.21 (7.11)	<0.0001	100	94.53 (6.39)	−5.47 (6.39)	<0.0001	−2.74 (−5.68 to 0.20)	0.067
Waist circumference, cm	100.10(9.42)	91.76 (12.02)	−8.35 (7.21)	<0.0001	99.55 (10.51)	94.50 (11.86)	−4.85 (6.53)	<0.0001	−3.49 (−6.83 to −0.5)	0.023
Body fat mass, kg	31.83 (9.06)	26.36 (8.14)	−5.40 (5.83)	<0.0001	33.02 (9.52)	29.07 (9.76)	−3.95 (5.98)	0.0006	−1.44 (−4.01 to 1.12)	0.266
Lean body mass, kg	28.70 (6.54)	27.84 (6.65)	−0.90 (1.38)	<0.0001	29.36 (8.41)	28.16 (6.39)	−1.44 (7.32)	<0.0001	0.55 (−1.8 to 2.89)	0.632
Percent body fat, %	37.93 (7.23)	34.31 (7.16)	−3.62 (4.12)	<0.0001	38.74 (5.66)	35.99 (6.39)	−2.75 (4.98)	0.001	−0.87 (−2.80 to 1.06)	0.386
Visceral fat area, cm^2^	121.46 (44.02)	93.18 (31.35)	−28.28 (32.08)	<0.0001	125.95 (43.79)	112.69 (38.88)	−13.26 (23.10)	0.004	−15.03 (−29.01 to −1.04)	0.043

All such values are presented as mean ± SE. Linear mixed-effect model adjusted to analyze the difference within each group. PSVLCD, protein-supplemented very-low-calorie diet program.

The authors additionally evaluated whether these trends were consistently observed across different BMI groups and genders through stratified analyses. Unfortunately, the reduction in the number of participants following stratification limited the statistical power of these analyses. As a result, the findings from the stratified analyses were presented in the Supplementary Table S4. As presented in the supplementary table, a significant reduction in waist circumference was observed among female participants (−9.61 ± 8.11 cm vs. −4.93 ± 6.52 cm, *p* = 0.0196), with marginal improvements in visceral fat area, body weight, and percentage of weight loss compared to the control group. When analyzed by BMI categories, the results revealed that in participants with a BMI < 30, the reduction in waist circumference was particularly pronounced. Conversely, in participants with a BMI ≥ 30, there were marginal improvements in weight loss percentage, body fat percentage, and visceral fat area compared to the control group.

During the 8-month weight maintenance phase (months 5–12), there were no significant differences between the groups with respect to any measures of change in obesity, except waist circumference and visceral fat area. Despite the reduction in body weight, percentage of weight loss from baseline, and waist circumference, lean body mass was marginally more preserved in the PSVLCD group (−0.90 ± 1.38 kg, *p* < 0.001) than in the control group (−1.44 ± 7.32 kg, *p* < 0.001), although there was no significant difference between the groups (*p* = 0.632).

### 3.3 Changes in cardiometabolic risk factors and bone mineral density

Table 3 and Supplementary Table S2 show the cardiometabolic risk factors between the PSVLCD and control groups. There was a significant reduction in systolic BP, diastolic BP, glucose level, HbA1c level, total cholesterol level, TG level, AST level, ALT level, and GGT level within each group at the completion of the study without any significant differences between the groups. Systolic BP decreased by −10.53 ± 14.87 mmHg (*p* < 0.001) in the PSVLCD group and only decreased by −6.61 ± 12.99 mmHg (*p* = 0.0023) in the control group. The glucose and total cholesterol levels decreased by −9.44 ± 11.72 mg/dL (*p* < 0.001) and − 95.23 ± 60.03 mg/dL (*p* < 0.001), respectively, in the PSVLCD group and decreased by −7.32 ± 12.98 mg/dL (*p* = 0.0008) and − 78.78 ± 60.73 mg/dL (*p* < 0.001), respectively, in the control group.

**Table 3:** Changes in cardiometabolic risk factors and bone mineral density.

Variables	PSVLCD group (*N* = 48)				Control group (*N* = 45)				Difference between groups (95% CI)	*p*-value
	Baseline	12 months	change	*p*-value	Baseline	12 months	change	*p*-value		
Systolic blood pressure, mmHg	130.17 (17.16)	120.05 (16.33)	−10.53 (14.87)	<0.0001	128.44 (14.33)	121.00 (12.33)	−6.61 (12.99)	0.0023	−3.93 (−10 to 2.15)	0.202
Diastolic blood pressure, mmHg	81.63 (12.12)	77.21 (11.71)	−4.58 (10.24)	0.0054	78.96 (9.10)	77.39 (8.98)	−1.02 (10.62)	0.5405	−3.56 (−8.09 to 0.97)	0.122
Glucose, mg/dL	105.31 (17.02)	95.74 (12.24)	−9.44 (11.72)	<0.0001	107.91 (20.13)	99.49 (17.30)	−7.32 (12.98)	0.0008	−2.12 (−7.49 to 3.24)	0.433
Glycated hemoglobin, %	5.60 (0.58)	5.46 (0.60)	−0.14 (0.28)	0.0022	5.83 (0.64)	5.61 (0.52)	−0.19 (0.30)	0.0002	0.05 (−0.08 to 0.18)	0.427
HOMA-IR	3.42 (3.26)	2.37 (2.84)	−0.88 (3.65)	0.1218	2.96 (1.29)	2.66 (1.94)	−0.28 (1.48)	0.2275	−0.60 (−1.8 to 0.61)	0.333
Total cholesterol, mg/dL	200.47(33.75)	202.56 (37.91)	2.09 (23.80)	0.567	191.32 (35.91)	193.54 (33.94)	2.22 (23.23)	0.544	−0.13(−10.31 to 10.05)	0.980
HDL cholesterol, mg/dL	53.98 (12.62)	56.79 (12.64)	2.56 (9.12)	0.0728	53.53 (10.93)	55.76 (12.05)	2.15 (8.20)	0.1016	0.41 (−3.36 to 4.18)	0.829
LDL cholesterol, mg/dL	128.04 (27.51)	131.35 (34.78)	4.16 (22.20)	0.0728	118.07 (32.28)	120.20 (32.87)	0.90 (22.52)	0.7988	3.26 (−6.45 to 12.97)	0.506
Triglyceride, mg/dL	122.40 (47.59)	105.23 (53.42)	−19.63 (45.93)	0.0076	125.69 (56.03)	112.54 (58.18)	−11.20 (45.73)	0.1249	−8.43 (−28.34 to 11.47)	0.402
AST, IU/dL	28.42 (18.13)	23.14 (10.01)	−5.37 (15.15)	0.025	26.51 (17.36)	23.68 (7.74)	−2.17 (16.56)	0.4061	−3.20 (−10.08 to 3.68)	0.358
ALT, IU/dL	42.42 (47.23)	28.00 (27.94)	−15.30 (38.63)	0.0129	34.13 (31.18)	26.07 (16.88)	−7.12 (31.46)	0.155	−8.18 (−23.52 to 7.16)	0.292
GGT, U/dL	32.94 (26.61)	25.12 (19.37)	−7.74 (21.86)	0.0251	33.04 (32.12)	27.90 (29.94)	−5.27 (20.63)	0.1099	−2.48 (−11.71 to 6.76)	0.595
Hepatic steatosis index	42.27 (5.29)	40.38 (4.99)	−1.85 (3.24)	<0.0001	42.14 (6.98)	41.07 (6.12)	−0.99 (3.20)	<0.0001	−0.87 (−2.26 to 0.53)	0.22
eGRF, mL/min/1.73m^2^	108.39 (12.82)	108.86 (12.43)	0.54 (7.62)	0.6459	107.22 (12.64)	106.16 (13.02)	−1.98 (6.26)	0.0494	2.52 (−0.52 to 5.55)	0.102
Uric acid, mg/dL	6.29 (1.90)	6.06 (1.75)	−0.30 (0.82)	0.3768	5.51 (1.19)	5.54 (1.34)	0.09 (0.82)	0.5052	−0.39 (−0.75 to −0.03)	0.033
Bone mineral density of the femoral neck, g/cm^2^	0.30 (1.24)	0.27 (1.20)	−0.08 (0.37)	0.1739	0.41 (1.59)	0.31 (1.56)	0.01 (0.16)	0.1058	0.01 (−0.15 to 0.16)	0.912
Bone mineral density of the lumbar spine, g/cm^2^	0.76 (1.01)	0.79 (1.07)	−0.01 (0.23)	0.6711	0.72 (1.04)	0.74 (1.07)	0.01 (0.16)	0.7908	−0.02 (−0.11 to 0.07)	0.621

All such values are presented as mean ± SE. Linear mixed-effect model adjusted to analyze the difference within each group. PSVLCD, protein-supplemented very-low-calorie diet program. HOMA-IR, homeostasis model of assessment of insulin resistance; eGFR, estimated glomerular filtration rate. HOMA-IR is calculated as the level of fasting glucose (measured in millimoles per liter) times the level of fasting insulin (measured in microunits per milliliter) divided by 22.5. Hepatic steatosis index is a screening tool reflecting nonalcoholic fatty liver disease and calculated using the following formula: 8 × (ALT/AST ratio) + BMI (+2, if female; +2, if diabetes mellitus). eGFR is calculated using the Chronic Kidney Disease Epidemiology Collaboration equation. Normal laboratory ranges are as follows: Glucose: 70–110 mg/dL; Total Cholesterol: 0–200 mg/dL; HDL Cholesterol: Men: 35–55 mg/dL, Women: 45–65 mg/dL; LDL Cholesterol: Men: 55–155 mg/dL, Women: 55–165 mg/dL; Triglycerides (TG): 0–150 mg/dL; AST: 0–40 IU/L; ALT: 0–40 IU/L; GGT: Men: 11–63 U/L, Women: 8–35 U/L; eGFR: >60 mL/min/1.73 m^2^.

At 12 months of the trial, the decrease in diastolic BP, TG level, AST level, ALT level, and GGT level was only observed in the PSVLCD group (−4.58 ± 10.24 mmHg, *p* = 0.054; −19.63 ± 45.93 mg/dL, *p* = 0.0076; −5.37 ± 15.15 mg/dL, *p* = 0.0250; −15.30 ± 38.63 mg/dL, *p* = 0.0129; and − 7.74 ± 21.86, *p* = 0.0251, respectively), and uric acid also significantly decreased until 8 months (−0.45 ± 0.83, *p* = 0.0008).

The calculated eGFR decreased only in the control group, but there was no clinical significance at the completion of the study (−1.98 ± 6.26 mg/mL, *p* = 0.0494). Moreover, there was no decrease in bone mineral density in either group.

### 3.4 Evaluation of the questionnaires

As shown in Table 4 and Supplementary Table S3, regarding the evaluation of dietary patterns, the overeating pattern increased in the control group compared with that in the PSVLCD group, and this difference was statistically significant (−0.64 ± 0.66 vs. -0.43 ± 0.70, *p* = 0.0435). However, high fat intake, nutritional imbalance, and impulsive eating did not differ significantly between the two groups. Although there were no significant differences between the groups, high fat intake slightly decreased at 4 and 8 months (−0.37 ± 0.56, *p* = 0.0035 and − 0.31 ± 0.41, *p* = 0.0141, respectively) and impulsive eating decreased at 12 months (−0.48 ± 0.72, *p* = 0.0266) in the PSVLCD group. Therefore, in the PSVLCD group, overeating, high fat intake, and impulsive eating improved compared with those in the control group.

**Table 4:** Effect of dietary intervention on the quality of life, quality of sleep, mood, and physical activities.

Variables	PSVLCD group (*N* = 48)				Control group (*N* = 45)				Difference between groups (95% CI)	*p*-value
	Baseline	12 months	change	*p*-value	Baseline	12 months	change	*p*-value		
GPAQ, median (IQR)	1250.10 (1774.88)	692.40 (887.07)	−228.20 (1057.70)	0.2914	1111.16 (1537.76)	843.33 (1232.92)	−447.14 (2446.94)	0.4123	218.94 (−961.13 to 1399.01)	0.687
Overeating	3.54 (0.59)	2.96 (0.73)	−0.64 (0.66)	0.003	3.26 (0.70)	2.39 (0.58)	−0.43 (0.70)	0.0715	−0.22 (−0.78 to −0.35)	0.0435
High fat intake	3.00 (0.76)	2.85 (0.65)	−0.26 (0.71)	0.204	2.83 (0.73)	2.39 (0.71)	−0.33 (0.59)	0.096	0.07 (−0.48 to 0.62)	0.789
Nutritional imbalance	3.04 (0.56)	3.17 (0.54)	0.01 (0.55)	0.924	2.99 (0.75)	3.02 (0.80)	0.07 (0.62)	0.7031	−0.06 (−0.54 to 0.42)	0.804
Impulsive eating	3.00 (0.84)	2.50 (0.52)	−0.48 (0.72)	0.027	2.68 (1.01)	1.88 (0.91)	−0.31 (0.67)	0.1579	−0.17 (−0.75 to 0.41)	0.551
HADS-anxiety	5.90 (3.63)	4.83 (3.73)	−0.52 (2.77)	0.227	5.62 (3.69)	4.76 (3.53)	−0.74 (3.21)	0.1655	0.21 (−1.12 to 1.54)	0.751
HADS-depression	7.40 (3.27)	5.17 (2.86)	−1.95 (2.85)	<0.0001	7.40 (3.85)	5.03 (3.98)	−2.38 (3.39)	<0.0001	0.43 (−0.95 to 1.81)	0.535
PSQI	5.94 (2.84)	4.98 (2.45)	−0.67 (2.13)	0.049	6.39 (3.37)	5.74 (2.54)	−0.89 (3.62)	0.1364	0.23 (−1.12 to 1.58)	0.729
KOQOL	40.78 (9.52)	34.07 (7.08)	−5.14 (9.05)	0.053	39.86 (9.95)	30.82 (12.19)	−4.36 (6.62)	0.0537	−0.78 (−7.52 to 5.96)	0.813

PSVLCD, protein-supplemented very-low-calorie diet program; GPAQ, Global Physical Activity Questionnaires; HADS, Hospital Anxiety and Depression Scale; PSQI, Pittsburg Sleep Quality Index; KOQOL, Korean version of Obesity-related Quality of Life Scale.

The physical activity levels, measured by the Global Physical Activity Questionnaire (GPAQ), showed a decrease over the 12-month period for both groups. The PSVLCD group had a median of 1250.10 (±1744.88) MET minutes/week at baseline, which decreased to 692.40 (±887.07) MET minutes/week after 12 months. The control group had a median of 1111.16 (±1537.76) MET minutes/week at baseline, which decreased to 843.33(±1232.92) MET minutes/week after 12 months. These findings indicate a reduction in overall physical activity in both groups.

The Hospital Anxiety and Depression Scale depression score decreased from baseline in both groups (−1.95 ± 2.85, *p* < 0.001 in the PSVLCD group vs. −2.38 ± 3.39, *p* < 0.001 in the control group). The Pittsburgh Sleep Quality Index (PSQI) value significantly decreased from 5.94 to 4.98 at 12 months (−0.67 ± 2.13, *p* = 0.0487) in the PSVLCD group, indicating an improvement in sleep quality, which is clinically significant since the cutoff for the PSQI is 5 (27). The Korean version of the Obesity-related Quality of Life Scale score significantly decreased at 4 and 8 months only in the PSVLCD group, indicating an improvement in the quality of life (−5.88 ± 9.59, *p* = 0.0064 and − 4.93 ± 7.48, *p* = 0.0283, respectively).

### 3.5 Adverse events from baseline to 12 months

Table 5 summarizes the side effects experienced by the participants in both groups. Thirty-two (67%) and 21 (47%) participants experienced adverse events in the PSVLCD and control groups, respectively. The PSVLCD group experienced a higher frequency of constipation, headache, dizziness, and myalgia than the control group. However, these manifestations were mild, transient, and mostly improved voluntarily. Some patients experienced gout, which was managed on an outpatient basis. Additionally, a few patients who dropped out of the study attributed their discontinuation to accompanying symptoms (32).

**Table 5:** Adverse events from baseline to 12 months.

	PSVLCD group	Control group	*p*-value
Number of serious adverse events	0	0	
Number of participants with any adverse events, *n* (%)	32 (67)	21 (47)	0.082
Total number of any adverse events, *n* (%)	83 (64)	46 (36)	0.048
Frequently reported adverse events, *n* (%)			
Constipation	12 (14)	3 (7)	
Headache	11 (13)	4 (9)	
Dizziness	8 (10)	3 (7)	
Myalgia	9 (11)	0 (0)	
Upper respiratory infection	4 (5)	4 (9)	
Indigestion	5 (6)	2 (4)	
Diarrhea	7 (8)	1 (2)	
Low back pain	4 (5)	3 (7)	
COVID	1 (1)	2 (4)	
Gout	1 (1)	1 (2)	

## 4 Discussion

A recent meta-analysis on the effectiveness of meal replacement-based diets compared with food-based caloric restriction also showed the superiority of the former, more than 60% of total daily energy intake from meal replacement resulted in the greatest effects (35). Herein, the PSVLCD group achieved a significant reduction in waist circumference throughout the study period. Moreover, PSVLCD was more effective than a calorie-restricted diet in inducing weight loss over 8 months. Additionally, there was a marginally significant difference in weight loss between PSVLCD and control groups at the end of the study. This phenomenon of the statistical significance being lost is not presumed to be due to unsuccessful weight loss in the PSVLCD group, rather due to the meaningful results of the active coaching intervention in the control group. The PSVLCD group lost 6.86 kg, achieving 91% of expected weight loss of 7.5 kg, while the control group lost 4.66 kg, achieving 186% of expected weight loss of 2.5 kg. Therefore, active online coaching through messenger programs could be a potential intervention for successful weight loss and weight-loss maintenance if users are motivated and ready to adhere to the program. The higher dropout rate in the control group, leading to the selective retention of those with stronger motivation, also explains the favorable results in the control group.

The PSVLCD group also exhibited greater reduction in body weight and fat mass, preserved lean body mass, and decreased TG and hepatic steatosis indices, although the differences were not statistically significant compared with the control group. These findings agree with those of a meta-analysis showing that calorie-restricted high-protein diet has beneficial effects on weight loss, body composition, resting energy expenditure, and TG levels (36).

Herein, PSVLCD led to rapid and intense weight reduction, with approximately three times higher weight and waist circumference reduction within 15 days. Although maximum loss (−7.89 kg) was observed at 8 months, the effects were maintained as evidenced by weight loss at 4 months (−7.70 kg) and subsequently at 6 and 12 months (−7.59 kg and − 6.86 kg, respectively). Previous qualitative reviews have shown that greater initial VLCD-induced weight loss is associated with greater long-term weight loss (28). Here, the PSVLCD group experienced higher initial weight loss than the control group, indicating a significant difference in the percentage of weight loss from baseline. In previous meta-analyses (20), VLCDs demonstrated excellent initial weight-loss effects. Most participants in VLCD programs do not regain much of the lost weight within 2–6 months and maintain at least some weight loss in the long term (37). Our participants underwent the VLCD program for 4 months; intermittent VLCD administration for a short period seemed to help maintain weight loss for at least another 4 months. However, after 8 months, the lost weight was not maintained. If PSVLCD can be combined with more evidence-based and effective supportive strategies, this program can be considered a viable option for long-term weight loss and maintenance.

Notably, the attrition rate in the PSVLCD group was 17%, half of that in the control group (Figure 1). Thus, providing meal replacement could improve adherence to the coaching program. A study on weight loss and dropout during a commercial weight-loss program also showed that a meal replacement-based VLCD group showed the greatest weight loss with the least dropout rate compared with an LCD or restricted normal food group (6).

Herein, VLCDs consisted of 600–800 calories during the initial 2 weeks of weight loss, and stepped food reintroduction was allowed after the initial 2 weeks. Most daily calories consumed by participants in this program were through low-calorie and very low-calorie diets. Thus, we only observed non-serious side effects such as constipation and fatigue, which were mild and easily managed. Nevertheless, it may be necessary to inform participants in advance of potential side effects that may occur more frequently than in the control group when using protein-supplemented meal replacement for weight loss.

High-protein meal replacements reduce body fat, preserve muscle mass, and increase satiety (38, 39). Protein’s superior satiety compared to carbs and fats, along with its ability to trigger fullness hormones, aids in reducing overall calorie intake, crucial for weight loss. This high satiety value also directly contributes to decreased appetite, leading to reductions in body weight and fat. Protein consumption increases concentration of satiety hormones and energy expenditure during metabolism, leading to increased satiety (40). Protein’s greater thermic effect requires more energy for digestion and metabolism than carbohydrates and fats, potentially boosting metabolic rate and aiding in weight loss. Additionally, a high-protein diet can help maintain lean body mass and prevent weight regain (41). During weight loss, there is a risk of losing lean body mass (muscle mass) along with fat mass. Adequate protein intake helps preserve lean body mass by providing the necessary amino acids for muscle repair and growth. Preserving muscle mass is important for maintaining metabolic rate and preventing the decrease in metabolic rate that often accompanies weight loss. A recent multicenter randomized controlled trial (RCT) showed that formula diet meal replacement induced greater weight loss than lifestyle interventions alone in patients with obesity and cardiovascular risk factors (42).

Overeating or impulsive food intake due to hunger is a challenge in managing patients with obesity (31). However, these dietary patterns improved in our PSVLCD group, possibly because of the high-protein content and bioavailable supplementation with essential amino acids. Furthermore, the PSVLCD group reported improved sleep quality and quality of life and maintained physical activity levels. Interactive online support with coaching, including continued behavioral therapy through online chats with dietitians and recommendations to use 1–2 meal replacements per day, also contributed to sustained weight loss. Similar to that reported in a previous meta-analysis (35), intensive lifestyle modification using KakaoTalk led to significant weight loss in both groups, supporting PSVLCD as an effective long-term weight-loss regimen without a negative impact on sleep or quality of life.

We observed a significant reduction in waist circumference in the PSVLCD group compared with in the control group. This parameter is an important diagnostic criterion for metabolic syndrome as an important risk factor for cardiovascular disease (36). Additionally, we also observed a similar reduction in visceral fat area measured by BIA. The InBody 770 already proved its comparability in estimating visceral fat area (25) as well as skeletal muscle mass estimation (24). We also measured correlation coefficient between waist circumference and visceral fat area and it showed 0.687, a moderate positive correlation.

During both weight loss and weight management phases, we observed a reduction in systolic BP, fasting glucose levels, HbA1c, and total cholesterol levels in both groups. However, diastolic BP, TG level, and liver enzyme marker (AST, ALT, and GGT) levels decreased only in the PSVLCD group. Early rapid weight loss can lead to T2DM remission by reducing liver fat, improving liver insulin sensitivity, decreasing pancreatic fat, and restoring early insulin secretion ability in beta cells (6, 37). Thus, the early rapid weight loss achieved through PSVLCD may have contributed to these improvements, similar to previous findings (28). The initial reductions in waist circumference, visceral fat area, and hepatic steatosis index observed in the PSVLCD group are indicative of significant improvements in central obesity, visceral fat accumulation, and liver health, all of which are closely associated with metabolic health. The decrease in waist circumference and visceral fat area indicates a reduction in central and visceral adiposity, both of which are critical in lowering the risk of metabolic and cardiovascular diseases. Additionally, the improvement in the hepatic steatosis index points to a significant positive impact on liver health, which is crucial for individuals with or at risk of metabolic dysfunction associated steatotic liver disease. Together, these changes imply that the PSVLCD diet could be a powerful tool in managing and potentially reversing metabolic syndrome and its associated risks.

The increase in LDL levels observed in both groups necessitates further investigation, especially considering the typical inverse relationship between BMI and cholesterol levels. Research indicates that LDL cholesterol can rise in response to carbohydrate-restricted diets even in contexts of low cardiometabolic risk (43). Similar findings have been reported in studies such as those by Sharman et al. (44) and research on the Atkins diet (45). In our study, factors contributing to the LDL increase may include variability in individual responses, issues with dietary adherence, physical activity and other metabolic factors. These aspects should be addressed in future research on dietary interventions and cholesterol management.

To our knowledge, this is the first RCT in South Korea with a large sample size and 1-year duration that provides a practical option of protein-supplemented calorie-restricted meal replacement with 30% daily protein intake for individuals with metabolic disorders and obesity. Moreover, intensive lifestyle modifications were personalized through online chats with one-on-one coaching by dietitians. The study was also conducted at two centers to minimize selection bias. In addition, our study may have clinical significance in the context that there have been limited studies conducted on East Asian populations, including among Koreans, who have different obesity standards compared to Western populations. In addition, one advantage of this study is the ethical consideration in not applying a placebo sachet to the control group, as the study’s objective was to ascertain the effects of PSVLCD, which is easily consumable, compared to maintaining a calorie-restricted diet.

This study has some limitations. First, the lack of active coaching or evaluation with limited exercise guidelines, combined with the absence of criteria for adherence assessment, could have impacted the interpretation of the results. Second, the lack of comparison between the protein types and precise quantitative assessments of protein intake in the control group against the intervention group may have compromised the study’s ability to establish a clear cause-and-effect relationship between the intervention and observed outcomes. Additionally, most of the adverse events were anticipated side effects of weight loss and were determined not to have affected the design or compliance of the study. It is acknowledged as a limitation that some patients may have experienced constraints in participating in the program due to anticipated side effects associated with weight loss. Third, the findings may not apply to individuals with diabetes or eating disorders as the study excluded participants with these conditions; therefore, caution is needed when generalizing results to these populations. Long-term weight-loss maintenance often requires third-wave cognitive behavioral therapy, which was not included (35). Fourth, the study duration and lack of long-term strategies showed significant weight-loss effects in the initial 4 months; however, beyond that period, participants tended to regain weight or experience reduced weight loss. Therefore, additional strategies are required to achieve long-term maintenance. Longer follow-up and incorporation of cognitive behavioral therapy would validate these results and enhance sustained weight loss. Furthermore, there is a need for future studies to address these methodological gaps for a more comprehensive understanding of the effects of protein interventions. Our study did not include other anthropometric measurements, such as midarm fold thickness, or functional assessments like hand grip strength. Beyond simple weight loss, further evaluation of muscle function and its potential impact on sarcopenia would provide valuable insights into how this program might influence conditions such as sarcopenic obesity, which has become a growing concern. If such assessments demonstrate positive effects, this program could be considered a potential strategy for managing this condition, balancing both the benefits and risks.

Additionally, the mean age of 43 years indicates an underrepresentation of older adults. Despite efforts to recruit participants evenly across age groups, the low participation rate from those aged 65 and older may affect the representativeness of the older age group in evaluating the program’s effectiveness.

Also our study did not include other anthropometric measurements including midarm fold thickness or functional assessments such as hand grip. Beyond simple weight loss, further evaluation of muscle function and the potential impact on sarcopenia would provide valuable insights into how this program might influence conditions such as sarcopenic obesity, which has become a growing concern. If such assessments demonstrate positive effects, this program could be considered as a potential prescription strategy, balancing both the benefits and risks for managing this condition.

Although it needs to be validated in future studies accompanied by long-term support strategies, PSVLCD could be an effective option for managing obesity in clinical, community, and healthcare settings. Especially considering that in East Asian populations, a BMI over 30 is considered obese, while in Western populations, the same BMI is classified as overweight, this study underscores the potential of PSVLCD as an effective weight-loss option in Western populations.

## 5 Conclusion

In this first RCT conducted in South Korea, the PSVLCD reduced waist circumference significantly, and a meaningful reduction was sustained throughout the study period, with a marginally significant decrease in weight loss. The PSVLCD significantly improved various health parameters, including blood pressure, blood glucose levels, HbA1c, total cholesterol levels, triglyceride levels, hepatic steatosis index, and overeating behavior. Additionally, it preserved lean body mass and enhanced the quality of life and sleep of participants without serious adverse events. Our study indicates that the PSVLCD, followed by a transition to a more sustainable low-calorie diet, could be a practical and effective long-term weight-loss strategy for individuals with obesity, metabolic disorders, and cardiovascular risk factors. This phased approach acknowledges the challenge of maintaining a strict VLCD indefinitely and offers a more feasible regimen for long-term adherence. Moreover, this study’s focus on an Asian population, where obesity diagnostic criteria and dietary habits differ from those in Western populations, underscores its significance. The findings suggest that the PSVLCD can be a safe and applicable weight-loss strategy tailored to the specific needs of this demographic. These insights contribute to the understanding of effective weight management strategies in diverse populations.

## Data Availability

The raw data supporting the conclusions of this article will be made available by the authors, without undue reservation.

## Footnotes

^1^ https://business.kakao.com/info/kakaotalkchannel/

## References

[1] World Health Organization. Obesity and overweight (2021) Available at: http://www.who.int/news-room/fact-sheets/detail/obesity-and-overweight (Accessed August 30, 2023).

[2] Seo MH Lee WY Kim SS Kang JH Kang JH Kim KK. 2018 Korean society for the study of obesity guideline for the management of obesity in Korea. J Obes Metab Syndr. (2019) 28:40–5. doi: 10.7570/jomes.2019.28.1.40, PMID: 31089578 PMC6484940

[3] Pan WH Yeh WT. How to define obesity? Evidence-based multiple action points for public awareness, screening, and treatment: an extension of Asian-Pacific recommendations. Asia Pac J Clin Nutr. (2008) 17:370–4. PMID: 18818155

[4] Noronha JC Nishi SK Braunstein CR Khan TA Blanco Mejia S Kendall CWC. The effect of liquid meal replacements on cardiometabolic risk factors in overweight/obese individuals with type 2 diabetes: a systematic review and meta-analysis of randomized controlled trials. Diabetes Care. (2019) 42:767–76. doi: 10.2337/dc18-2270, PMID: 30923163

[5] Astbury NM Piernas C Hartmann-Boyce J Lapworth S Aveyard P Jebb SA. A systematic review and meta-analysis of the effectiveness of meal replacements for weight loss. Obes Rev. (2019) 20:569–87. doi: 10.1111/obr.12816, PMID: 30675990 PMC6849863

[6] Juray S Axen KV Trasino SE. Remission of type 2 diabetes with very low-calorie diets—a narrative review. Nutrients. (2021) 13:2086. doi: 10.3390/nu13062086, PMID: 34207117 PMC8234895

[7] Lean MEJ Leslie WS Barnes AC Brosnahan N Thom G McCombie L. Durability of a primary care-led weight-management intervention for remission of type 2 diabetes: 2-year results of the DiRECT open-label, cluster-randomised trial. Lancet Diabetes Endocrinol. (2019) 7:344–55. doi: 10.1016/S2213-8587(19)30068-3, PMID: 30852132

[8] Lean ME Leslie WS Barnes AC Brosnahan N Thom G McCombie L. Primary care-led weight management for remission of type 2 diabetes (DiRECT): an open-label, cluster-randomised trial. Lancet. (2018) 391:541–51. doi: 10.1016/S0140-6736(17)33102-1 29221645

[9] Ardavani A Aziz H Smith K Atherton PJ Phillips BE Idris I. The effects of very low energy diets and low energy diets with exercise training on skeletal muscle mass: a narrative review. Adv Ther. (2021) 38:149–63. doi: 10.1007/s12325-020-01562-0, PMID: 33211298 PMC7854408

[10] Hsu KJ Liao CD Tsai MW Chen CN. Effects of exercise and nutritional intervention on body composition, metabolic health, and physical performance in adults with sarcopenic obesity: a meta-analysis. Nutrients. (2019) 11:2163. doi: 10.3390/nu11092163, PMID: 31505890 PMC6770949

[11] Dong JY Zhang ZL Wang PY Qin LQ. Effects of high-protein diets on body weight, glycaemic control, blood lipids and blood pressure in type 2 diabetes: meta-analysis of randomised controlled trials. Br J Nutr. (2013) 110:781–9. doi: 10.1017/S0007114513002055, PMID: 23829939

[12] Santesso N Akl EA Bianchi M Mente A Mustafa R Heels-Ansdell D. Effects of higher- versus lower-protein diets on health outcomes: a systematic review and meta-analysis. Eur J Clin Nutr. (2012) 66:780–8. doi: 10.1038/ejcn.2012.37, PMID: 22510792 PMC3392894

[13] Moon J Koh G. Clinical evidence and mechanisms of high-protein diet-induced weight loss. J Obesity Metab Syndrome. (2020) 29:166–73. doi: 10.7570/jomes20028, PMID: 32699189 PMC7539343

[14] Clifton PM Condo D Keogh JB. Long term weight maintenance after advice to consume low carbohydrate, higher protein diets–a systematic review and meta analysis. Nutr Metab Cardiovasc Dis. (2014) 24:224–35. doi: 10.1016/j.numecd.2013.11.006 24472635

[15] Parretti HM Jebb SA Johns DJ Lewis AL Christian-Brown AM Aveyard P. Clinical effectiveness of very-low-energy diets in the management of weight loss: a systematic review and meta-analysis of randomized controlled trials. Obes Rev. (2016) 17:225–34. doi: 10.1111/obr.12366, PMID: 26775902

[16] Magkos F. The role of dietary protein in obesity. Rev Endocr Metab Disord. (2020) 21:329–40. doi: 10.1007/s11154-020-09576-3 32740867

[17] Chaiyasoot K Sarasak R Pheungruang B Dawilai S Pramyothin P Boonyasiri A. Evaluation of a 12-week lifestyle education intervention with or without partial meal replacement in Thai adults with obesity and metabolic syndrome: a randomised trial. Nutr Diabetes. (2018) 8:23. doi: 10.1038/s41387-018-0034-0, PMID: 29695706 PMC5916885

[18] Gulati S Misra A Tiwari R Sharma M Pandey RM Yadav CP. Effect of high-protein meal replacement on weight and cardiometabolic profile in overweight/obese Asian Indians in North India. Br J Nutr. (2017) 117:1531–40. doi: 10.1017/S0007114517001295, PMID: 28653586

[19] Kuriyan R Lokesh DP D’souza N Priscilla DJ Peris CH Selvam S. Portion controlled ready-to-eat meal replacement is associated with short term weight loss: a randomised controlled trial. Asia Pac J Clin Nutr. (2017) 26:1055–65. 28917231 10.6133/apjcn.022017.07

[20] Xu DF Sun JQ Chen M Chen YQ Xie H Sun W-J. Effects of lifestyle intervention and meal replacement on glycaemic and body-weight control in Chinese subjects with impaired glucose regulation: a 1-year randomised controlled trial. Br J Nutr. (2013) 109:487–92. doi: 10.1017/S0007114512001328, PMID: 23021205

[21] Jo E Worts PR Elam ML Brown AF Khamoui AV Kim D-H. Resistance training during a 12-week protein supplemented VLCD treatment enhances weight-loss outcomes in obese patients. Clin Nutr. (2019) 38:372–82. doi: 10.1016/j.clnu.2017.12.015, PMID: 29352654

[22] Kim JY. Optimal diet strategies for weight loss and weight loss maintenance. J Obesity Metab Syndrome. (2021) 30:20–31. doi: 10.7570/jomes20065, PMID: 33107442 PMC8017325

[23] World Health Organization. The Asia-Pacific perspective: Redefining obesity and its treatment. Geneva: World Health Organization (2000).

[24] Lee SY Ahn S Kim YJ Ji MJ Kim KM Choi SH. Comparison between dual-energy X-ray absorptiometry and bioelectrical impedance analyses for accuracy in measuring whole body muscle mass and appendicular skeletal muscle mass. Nutrients. (2018) 10:738. doi: 10.3390/nu10060738 29880741 PMC6024648

[25] Park KS Lee DH Lee J Kim YJ Jung KY Kim KM. Comparison between two methods of bioelectrical impedance analyses for accuracy in measuring abdominal visceral fat area. J Diabetes Complicat. (2016) 30:343–9. doi: 10.1016/j.jdiacomp.2015.10.014, PMID: 26620129

[26] Levey AS Stevens LA Schmid CH Zhang YL Castro AF III Feldman HI. A new equation to estimate glomerular filtration rate. Ann Intern Med. (2009) 150:604–12. doi: 10.7326/0003-4819-150-9-200905050-00006, PMID: 19414839 PMC2763564

[27] Lee JH Kim D Kim HJ Lee CH Yang JI Kim W. Hepatic steatosis index: a simple screening tool reflecting nonalcoholic fatty liver disease. Dig Liver Dis. (2010) 42:503–8. doi: 10.1016/j.dld.2009.08.002, PMID: 19766548

[28] Matthews DR Hosker JP Rudenski AS Naylor BA Treacher DF Turner RC. Homeostasis model assessment: insulin resistance and β-cell function from fasting plasma glucose and insulin concentrations in man. Diabetologia. (1985) 28:412–9. doi: 10.1007/BF00280883, PMID: 3899825

[29] Rachner TD Khosla S Hofbauer LC. Osteoporosis: now and the future. Lancet. (2011) 377:1276–87. doi: 10.1016/S0140-6736(10)62349-5, PMID: 21450337 PMC3555696

[30] Park HS Sung SW Ou SW Lee KY Kim BS Han JH. Development of Korean version of obesity-related quality of life scale. J Kor Soc Study Obes. (2003) 12:280–93.

[31] Do Lee YD Kim KW Choi KS Kim M Cho YJ Sohn C. Development of dietary pattern evaluation tool for adults and correlation with dietary quality index. Nutr Res Pract. (2016) 10:305–12. doi: 10.4162/nrp.2016.10.3.305, PMID: 27247727 PMC4880730

[32] Lee J Lee C Min J Kang DW Kim JY Yang HI. Development of the Korean global physical activity questionnaire: reliability and validity study. Glob Health Promot. (2020) 27:44–55. doi: 10.1177/1757975919854301, PMID: 31375056

[33] Choi JH Jeon S Hong S Kim A Park JY Yang HJ. The reliability and validity of the Korean version of hospital anxiety and depression scale using Rasch measurement theory in patients with Parkinson’s disease. J Korean Neurol Assoc. (2021) 39:312–21. doi: 10.17340/jkna.2021.4.7

[34] Sohn SI Kim DH Lee MY Cho YW. The reliability and validity of the Korean version of the Pittsburgh sleep quality index. Sleep Breath. (2012) 16:803–12. doi: 10.1007/s11325-011-0579-9, PMID: 21901299

[35] Min J Kim SY Shin IS Park YB Lim YW. The effect of meal replacement on weight loss according to calorie-restriction type and proportion of energy intake: a systematic review and meta-analysis of randomized controlled trials. J Acad Nutr Diet. (2021) 121:1551–1564.e3. doi: 10.1016/j.jand.2021.05.001 34144920

[36] Wycherley TP Moran LJ Clifton PM Noakes M Brinkworth GD. Effects of energy-restricted high-protein, low-fat compared with standard-protein, low-fat diets: a meta-analysis of randomized controlled trials. Am J Clin Nutr. (2012) 96:1281–98. doi: 10.3945/ajcn.112.044321, PMID: 23097268

[37] Mulholland Y Nicokavoura E Broom J Rolland C. Very-low-energy diets and morbidity: a systematic review of longer-term evidence. Br J Nutr. (2012) 108:832–51. doi: 10.1017/S0007114512001924, PMID: 22800763

[38] Miller PE Alexander DD Perez V. Effects of whey protein and resistance exercise on body composition: a meta-analysis of randomized controlled trials. J Am Coll Nutr. (2014) 33:163–75. doi: 10.1080/07315724.2013.875365, PMID: 24724774

[39] Heymsfield SB Van Mierlo CA Van der Knaap HC Heo M Frier HI. Weight management using a meal replacement strategy: meta and pooling analysis from six studies. Int J Obes Relat Metab Disord. (2003) 27:537–49. doi: 10.1038/sj.ijo.0802258 12704397

[40] Morell P Fiszman S. Revisiting the role of protein-induced satiation and satiety. Food Hydrocoll. (2017) 68:199–210. doi: 10.1016/j.foodhyd.2016.08.003

[41] Lejeune MP Kovacs EM Westerterp-Plantenga MS. Additional protein intake limits weight regain after weight loss in humans. Br J Nutr. (2005) 93:281–9. doi: 10.1079/BJN20041305 15788122

[42] Halle M Röhling M Banzer W Braumann KM Kempf K McCarthy D. Meal replacement by formula diet reduces weight more than a lifestyle intervention alone in patients with overweight or obesity and accompanied cardiovascular risk factors-the ACOORH trial. Eur J Clin Nutr. (2021) 75:661–9. doi: 10.1038/s41430-020-00783-4 33128036

[43] Norwitz NG Feldman D Soto-Mota A Kalayjian T Ludwig DS. Elevated LDL cholesterol with a carbohydrate-restricted diet: evidence for a “lean mass hyper-responder” phenotype. Current Developments in Nutrition. (2022) 6:nzab144. doi: 10.1093/cdn/nzab144 35106434 PMC8796252

[44] Sharman MJ Kraemer WJ Love DM Avery NG Gomez AL Scheett TP. A ketogenic diet favorably affects serum biomarkers for cardiovascular disease in normal-weight men. J Nutr. (2002) 132:1879–85. doi: 10.1093/jn/132.7.1879, PMID: 12097663

[45] Yancy WS Olsen MK Guyton JR Bakst RP Westman EC. A low-carbohydrate, ketogenic diet versus a low-fat diet to treat obesity and hyperlipidemia: a randomized, controlled trial. Ann Intern Med. (2004) 140:769–77. doi: 10.7326/0003-4819-140-10-200405180-00006, PMID: 15148063

