# Supplementary material for: Effectiveness of a protein-supplemented very-low-calorie diet program for weight loss: a randomized controlled trial in South Korea
Source: Front Nutr. 2024 Sep 12;11:1370737. doi: 10.3389/fnut.2024.1370737 (PMC11424548; doi:10.3389/fnut.2024.1370737)
Supplement: Supplementary file 1 [file Table_1.docx]

**Supplementary Tables**

**Supplemental Table 1.** Changes in weight and body composition with the diet intervention including all intermediate time points.

| **Variables** | **PSVLCD group (N = 48)** | **p-value** | **Control group (N = 45)** | **p-value** | **Difference between groups (95% CI)** | **p-value** |  |
| --- | --- | --- | --- | --- | --- | --- | --- |
| **Body weight difference from baseline, kg** |  |  |  |  |  |  |  |
| 0.5 months | -4.08 (1.68) | <0.0001 | -1.82 (1.54) | <0.0001 | -2.25 (-2.92 to 1.59) | <0.001 |  |
| 2 months | -6.61 (3.70) | <0.0001 | -4.05 (2.89) | <0.0001 | -2.57 (-3.94 to 1.19) | <0.001 |  |
| 4 months | -7.70 (5.33) | <0.0001 | -5.36 (4.26) | <0.0001 | -2.33 (-4.33 to 0.34) | 0.022 |  |
| 6 months | -7.59 (5.83) | <0.0001 | -5.55 (5.15) | <0.0001 | -2.04 (-4.33 to 0.26) | 0.082 |  |
| 8 months | -7.89 (6.12) | <0.0001 | -5.45 (5.42) | <0.0001 | -2.44 (-4.92 to 0.04) | 0.054 |  |
| 10 months | -7.13 (6.63) | <0.0001 | -4.91 (5.36) | <0.0001 | -2.22 (-4.84 to 0.41) | 0.097 |  |
| 12 months | -6.86 (6.83) | <0.0001 | -4.66 (5.50) | <0.0001 | -2.20 (-4.9 to 0.5) | 0.109 |  |
| **Percentage of weight loss from baseline, %** |  |  |  |  |  |  |  |
| 0.5 months | -4.92 (1.84) | <0.0001 | -2.13 (1.77) | <0.0001 | -2.79 (-3.54 to 2.05) | <0.001 |  |
| 2 months | -7.93 (4.01) | <0.0001 | -4.61 (2.95) | <0.0001 | -3.32 (-4.76 to 1.87) | <0.001 |  |
| 4 months | -9.23 (5.67) | <0.0001 | -6.08 (4.45) | <0.0001 | -3.15 (-5.26 to 1.04) | 0.004 |  |
| 6 months | -9.16 (6.18) | <0.0001 | -6.23 (5.49) | <0.0001 | -2.93 (-5.37 to 0.49) | 0.019 |  |
| 8 months | -9.47 (6.48) | <0.0001 | -6.22 (6.08) | <0.0001 | -3.25 (-5.95 to 0.55) | 0.019 |  |
| 10 months | -8.53 (6.95) | <0.0001 | -5.76 (6.15) | <0.0001 | -2.77 (-5.62 to 0.09) | 0.057 |  |
| 12 months | -8.21 (7.11) | <0.0001 | -5.47 (6.39) | <0.0001 | -2.74 (-5.68 to 0.20) | 0.067 |  |
| **Waist circumference, cm** | |  |  |  |  |  |  |
| 0.5 months | | -3.35 (2.68) | <0.0001 | -1.04 (3.18) | 0.0724 | -2.31 (-3.70 to 1.11) | <0.001 |
| 2 months | | -6.08 (3.40) | <0.0001 | -2.99 (3.67) | <0.0001 | -3.09 (-4.85 to 1.63) | <0.001 |
| 4 months | | -7.27 (4.64) | <0.0001 | -4.40 (4.38) | <0.0001 | -2.87 (-5.13 to 1.01) | 0.003 |
| 6 months | | -7.18 (5.05) | <0.0001 | -4.41 (5.28) | <0.0001 | -2.77 (-5.23 to 0.62) | 0.012 |
| 8 months | | -9.35 (9.47) | <0.0001 | -4.74 (6.08) | <0.0001 | -4.61 (-8.47 to 1.2) | 0.009 |
| 10 months | | -7.62 (5.27) | <0.0001 | -4.88 (6.29) | <0.0001 | -2.75 (-5.38 to 0.2) | 0.035 |
| 12 months | | -8.35 (7.21) | <0.0001 | -4.85 (6.53) | <0.0001 | -3.49 (-6.83 to 0.5) | 0.023 |
| **Visceral fat area, cm^2^** | |  |  |  |  |  |  |
| 0.5 months | | -11.51 (11.22) | <0.0001 | -4.72 (11.78) | 0.0307 | -6.79 (-12.25 to -1.33) | 0.019 |
| 2 months | | -24.69 (20.18) | <0.0001 | -13.78 (20.51) | 0.0006 | -10.91 (-20.57 to -1.24) | 0.033 |
| 4 months | | -29.48 (22.15) | <0.0001 | -22.25 (16.50) | <0.0001 | -7.24 (-16.56 to 2.09) | 0.139 |
| 6 months | | -32.28 (27.11) | <0.0001 | -15.01 (22.99) | 0.0010 | -17.28 (-29.44 to -5.12) | 0.008 |
| 8 months | | -29.15 (31.92) | <0.0001 | -14.87 (18.93) | 0.0001 | -14.33 (-27.13 to -1.43) | 0.037 |
| 10 months | | -24.76 (31.96) | 0.0004 | -12.36 (26.11) | 0.0209 | -12.40 (-27.68 to 2.87) | 0.124 |
| 12 months | | -28.28 (32.08) | 0.0001 | -13.26 (23.10) | <0.0001 | -15.03 (-29.01 to -1.04) | 0.043 |
| **Body fat percent, %** |  |  |  |  |  |  |  |
| 0.5 months | -0.48 (1.59) | 0.041 | -0.24 (2.43) | 0.516 | -0.25 (-1.09 to 0.60) | 0.561 |  |
| 2 months | -3.21 (2.47) | <0.0001 | -1.70 (3.26) | 0.0021 | -1.52 (-2.71 to -0.32) | 0.013 |  |
| 4 months | -3.87 (3.72) | <0.0001 | -2.49 (3.96) | 0.0006 | -1.38 (-2.97 to 0.21) | 0.087 |  |
| 6 months | -4.07 (4.16) | <0.0001 | -2.67 (4.62) | 0.0004 | -1.41 (-3.19 to 0.38) | 0.129 |  |
| 8 months | -3.91 (4.04) | <0.0001 | -2.70 (4.55) | 0.0004 | -1.21 (-3.00 to 0.57) | 0.192 |  |
| 10 months | -3.46 (4.07) | <0.0001 | -2.66 (4.72) | 0.001 | -0.79 (-2.68 to 1.09) | 0.418 |  |
| 12 months | -3.62 (4.12) | <0.0001 | -2.75 (4.98) | 0.001 | -0.87 (-2.80 to 1.06) | 0.386 |  |
| **Body fat mass, kg** |  |  |  |  |  |  |  |
| 0.5 months | -2.02 (1.57) | <0.0001 | -0.91 (2.45) | 0.0296 | -1.11 (-1.97 to 0.25) | 0.010 |  |
| 2 months | -4.99 (3.15) | <0.0001 | -3.55 (5.38) | 0.0009 | -1.43 (-3.27 to 0.41) | 0.118 |  |
| 4 months | -5.84 (4.60) | <0.0001 | -4.10 (4.90) | <0.0001 | -1.74 (-3.7 to 0.22) | 0.081 |  |
| 6 months | -5.93 (5.45) | <0.0001 | -4.28 (5.73) | 0.0001 | -1.66 (-3.99 to 0.67) | 0.160 |  |
| 8 months | -6.00 (5.46) | <0.0001 | -4.25 (5.78) | 0.0005 | -1.75 (-4.16 to 0.66) | 0.153 |  |
| 10 months | -5.38 (5.77) | <0.0001 | -3.79 (5.52) | 0.001 | -1.59 (-4.08 to 0.89) | 0.205 |  |
| 12 months | -5.40 (5.83) | <0.0001 | -3.95 (5.98) | 0.0006 | -1.44 (-4.01 to 1.12) | 0.266 |  |
| **Skeletal muscle mass, kg** |  |  |  |  |  |  |  |
| 0.5 months | -1.21 (1.10) | <0.0001 | -1.40 (7.07) | <0.0001 | 0.19 (-1.96 to 2.33) | 0.855 |  |
| 2 months | -1.05 (1.03) | <0.0001 | -1.53 (7.09) | <0.0001 | 0.48 (-1.67 to 2.63) | 0.645 |  |
| 4 months | -1.29 (1.70) | <0.0001 | -1.70 (6.88) | <0.0001 | 0.41 (-1.71 to 2.53) | 0.691 |  |
| 6 months | -1.05 (1.17) | <0.0001 | -1.70 (6.94) | <0.0001 | 0.64 (-1.49 to 2.78) | 0.532 |  |
| 8 months | -1.18 (1.15) | <0.0001 | -1.67 (7.14) | 0.1402 | 0.49 (-1.76 to 2.74) | 0.654 |  |
| 10 months | -0.65 (2.77) | <0.0001 | -1.41 (7.38) | <0.0001 | 0.76 (-1.74 to 3.25) | 0.538 |  |
| 12 months | -0.90 (1.38) | <0.0001 | -1.44 (7.32) | <0.0001 | 0.55(-1.8 to 2.89) | 0.632 |  |

PSVLCD, protein-supplemented very-low-calorie diet program

**Supplemental Table 2.** Changes in cardiometabolic risk factors and bone mineral density during 12 months of the trial including all intermediate time points.

.

| Variables | **PSVLCD group (N = 48)** | **p-value** | **Control group (N = 45)** | **p-value** | **Difference between groups (95% CI)** | **p-value** |
| --- | --- | --- | --- | --- | --- | --- |
| **Systolic blood pressure, mmHg** |  |  |  |  |  |  |
| 4 months | -11.73 (13.29) | <0.0001 | -6.31 (11.94) | 0.0009 | -5.42 (-10.63 to 0.2) | 0.042 |
| 8 months | -12.09 (14.54) | <0.0001 | -4.05 (13.89) | 0.066 | -8.04 (-14.14 to 1.94) | 0.01 |
| 12 months | -10.53 (14.87) | <0.0001 | -6.61 (12.99) | 0.0023 | -3.93 (-10 to 2.15) | 0.202 |
| **Diastolic blood pressure, mmHg** |  |  |  |  |  |  |
| 4 months | -5.90 (10.65) | 0.0004 | -1.44 (9.03) | 0.2891 | -4.45 (-8.53 to 0.37) | 0.033 |
| 8 months | -3.95 (10.48) | 0.0162 | -0.02 (10.89) | 0.9888 | -3.93 (-8.51 to 0.65) | 0.092 |
| 12 months | -4.58 (10.24) | 0.0054 | -1.02 (10.62) | 0.5405 | -3.56 (-8.09 to 0.97) | 0.122 |
| **Glucose, mg/dL** |  |  |  |  |  |  |
| 4 months | -6.73 (12.72) | 0.0006 | -8.40 (14.56) | 0.0004 | 1.67 (-3.95 to 7.29) | 0.556 |
| 8 months | -7.32 (10.11) | <0.0001 | -6.60 (14.32) | 0.0048 | -0.72 (-6.07 to 4.63) | 0.787 |
| 12 months | -9.44 (11.72) | <0.0001 | -7.32 (12.98) | 0.0008 | -2.12 (-7.49 to 3.24) | 0.433 |
| **Glycated hemoglobin, %** |  |  |  |  |  |  |
| 4 months | -0.09 (0.33) | 0.0783 | -0.20 (0.38) | 0.0009 | 0.12 (-0.03 to 0.26) | 0.117 |
| 8 months | -0.14 (0.24) | 0.0004 | -0.17 (0.34) | 0.0026 | 0.03 (-0.1 to 0.16) | 0.659 |
| 12 months | -0.14 (0.28) | 0.0022 | -0.19 (0.30) | 0.0002 | 0.05 (-0.08 to 0.18) | 0.427 |
| **HOMA-IR** |  |  |  |  |  |  |
| 12 months | -0.88 (3.65) | 0.1218 | -0.28 (1.48) | 0.2275 | -0.60 (-1.8 to 0.61) | 0.333 |
| **Total cholesterol, mg/dL** |  |  |  |  |  |  |
| 4 months | -4.98 (22.85) | 0.1606 | -5.29 (20.54) | 0.1068 | 0.32 (-8.98 to 9.61) | 0.9471 |
| 8 months | -2.23 (22.32) | 0.5155 | -0.68 (20.12) | 0.8290 | -1.55 (-10.64 to 7.54) | 0.7395 |
| 12 months | 2.09 (23.80) | 0.5673 | 2.22 (23.23) | 0.5441 | -0.13 (-10.31 to 10.05) | 0.9804 |
| **HDL-cholesterol, mg/dL** |  |  |  |  |  |  |
| 4 months | -0.77 (7.80) | 0.4971 | 0.20 (8.65) | 0.8774 | -0.97 (-4.36 to 2.42) | 0.571 |
| 8 months | 2.73 (9.05) | 0.0521 | 2.95 (10.29) | 0.0701 | -0.23 (-4.38 to 3.93) | 0.914 |
| 12 months | 2.56 (9.12) | 0.0728 | 2.15 (8.20) | 0.1016 | 0.41 (-3.36 to 4.18) | 0.829 |
| **LDL-cholesterol, mg/dL** |  |  |  |  |  |  |
| 4 months | -2.65 (22.04) | 0.4097 | -1.58 (18.21) | 0.5640 | -1.07 (-9.42 to 7.29) | 0.8 |
| 8 months | -0.64 (22.72) | 0.0521 | -0.74 (20.33) | 0.8152 | 0.10 (-9.16 to 9.36) | 0.983 |
| 12 months | 4.16 (22.20) | 0.0728 | 0.90 (22.52) | 0.7988 | 3.26 (-6.45 to 12.97) | 0.506 |
| **Triglyceride, mg/dL** |  |  |  |  |  |  |
| 4 months | -29.52 (43.25) | <0.0001 | -22.84 (46.40) | 0.0019 | -6.68 (-25.14 to 11.79) | 0.474 |
| 8 months | -17.77 (44.80) | 0.0118 | -16.29 (45.44) | 0.0252 | -1.49 (-20.84 to 17.87) | 0.879 |
| 12 months | -19.63 (45.93) | 0.0076 | -11.20 (45.73) | 0.1249 | -8.43 (-28.34 to 11.47) | 0.402 |
| **AST, mg/dL** |  |  |  |  |  |  |
| 4 months | -6.10 (14.05) | 0.0042 | 1.53 (23.91) | 0.6692 | -7.64 (-15.82 to 0.54) | 0.062 |
| 8 months | -4.93 (13.98) | 0.0240 | -3.12 (16.41) | 0.2252 | -1.81 (-8.34 to 4.71) | 0.582 |
| 12 months | -5.37 (15.15) | 0.0250 | -2.17 (16.56) | 0.4061 | -3.20 (-10.08 to 3.68) | 0.358 |
| **ALT, mg/dL** |  |  |  |  |  |  |
| 4 months | -17.23 (36.41) | 0.002 | -1.93 (37.77) | 0.7329 | -15.3 (-30.58 to 0.02) | 0.05 |
| 8 months | -15.82 (35.65) | 0.0052 | -8.50 (30.23) | 0.0757 | -7.32 (-21.52 to 6.89) | 0.309 |
| 12 months | -15.30 (38.63) | 0.0129 | -7.12 (31.46) | 0.1550 | -8.18 (-23.52 to 7.16) | 0.292 |
| **GGT, mg/dL** |  |  |  |  |  |  |
| 4 months | -9.10 (23.35) | 0.0096 | -3.18 (28.06) | 0.4515 | -5.93 (-16.53 to 4.68) | 0.270 |
| 8 months | -8.41 (19.82) | 0.0073 | -5.81 (21.06) | 0.0811 | -2.60 (-11.36 to 6.17) | 0.557 |
| 12 months | -7.74 (21.86) | 0.0251 | -5.27 (20.63) | 0.1099 | -2.48 (-11.71 to 6.76) | 0.595 |
| **Hepatic steatosis index** |  |  |  |  |  |  |
| 4 months | -6.24 (4.33) | <0.0001 | -4.81 (3.67) | <0.0001 | -0.77 (-2.06 to 0.52) | 0.237 |
| 8 months | -6.57 (4.21) | <0.0001 | -4.46 (3.52) | <0.0001 | -1.32 (-2.52 to 0.11) | 0.032 |
| 12 months | -5.63 (4.89) | <0.0001 | -4.09 (4.48) | <0.0001 | -0.87 (-2.26 to 0.53) | 0.22 |
| **eGRF, mg/mL** |  |  |  |  |  |  |
| 4 months | -0.05 (7.06) | 0.9632 | 0.71 (5.82) | 0.4192 | -0.75 (-3.43 to 1.92) | 0.577 |
| 8 months | 0.96 (6.09) | 0.3013 | 0.40 (7.21) | 0.7220 | 0.56 (-2.29 to 3.42) | 0.696 |
| 12 months | 0.54 (7.62) | 0.6459 | -1.98 (6.26) | 0.0494 | 2.52 (-0.52 to 5.55) | 0.102 |
| **Uric acid, mg/dL** |  |  |  |  |  |  |
| 4 months | -0.26 (0.89) | 0.0472 | -0.06 (0.79) | 0.6339 | -0.2 (-0.55 to 0.14) | 0.248 |
| 8 months | -0.45 (0.83) | 0.0008 | -0.11 (0.76) | 0.3632 | -0.34 (-0.69 to 0) | 0.051 |
| 12 months | -0.30 (0.82) | 0.3768 | 0.09 (0.82) | 0.5052 | -0.39 (-0.75 to 0.03) | 0.033 |
| **Femoral neck, g/cm^2^** |  |  |  |  |  |  |
| 12 months | -0.08 (0.37) | 0.1739 | 0.01 (0.16) | 0.1058 | 0.01 (-0.15 to 0.16) | 0.912 |
| **Lumbar spine, g/cm^2^** |  |  |  |  |  |  |
| 12 months | -0.01 (0.23) | 0.6711 | 0.01 (0.16) | 0.7908 | -0.02 (-0.11 to 0.07) | 0.621 |

PSVLCD, protein-supplemented very-low-calorie diet program; HOMA-IR, homeostasis model of assessment of insulin resistance; eGFR, estimated glomerular filtration rate

HOMA-IR is calculated as the level of fasting glucose (measured in millimoles per liter) times the level of fasting insulin (measured in microunits per milliliter) divided by 22.5.

Hepatic steatosis index is a screening tool reflecting nonalcoholic fatty liver disease and is calculated using the following formula: 8 × (ALT/AST ratio) + BMI (+2, if female; +2, if diabetes mellitus).

eGFR using calculated by the Chronic Kidney Disease Epidemiology Collaboration equation.

**Supplemental Table 3.** Effect of dietary intervention on the quality of life, quality of sleep, mood, and physical activities including all intermediate time points.

| Variables | **PSVLCD group (N = 48)** | **p-value** | **Control group (N = 45)** | **p-value** | **Difference between groups (95% CI)** | **p-value** |
| --- | --- | --- | --- | --- | --- | --- |
| **GPAQ, median (IQR)** |  |  |  |  |  |  |
| 4 months | -473.54 (2063.35) | 0.1185 | -469.71 (1602.87) | 0.0557 | -3.83 (-768.42 to 760.76) | 0.992 |
| 8 months | -553.72 (2023.08) | 0.0799 | -824.10 (1636.83) | 0.0032 | 270.38 (-543.59 to 1084.35) | 0.510 |
| 12 months | -228.20 (1057.70) | 0.2914 | -447.14 (2446.94) | 0.4123 | 218.94 (-961.13 to 1399.01) | 0.687 |
| **Dietary pattern evaluation** |  |  |  |  |  |  |
| Overeating |  |  |  |  |  |  |
| 4 months | -0.49 (0.65) | 0.0012 | -0.34 (0.64) | 0.0471 | -0.14 (-0.56 to 0.28) | 0.492 |
| 8 months | -0.61 (0.41) | 0.0004 | -0.21 (0.84) | 0.4287 | -0.40 (-0.95 to 0.15) | 0.150 |
| 12 months | -0.64 (0.66) | 0.0028 | -0.43 (0.70) | 0.0715 | -0.22 (-0.78 to 0.35) | 0.0435 |
| **High fat intake** |  |  |  |  |  |  |
| 4 months | -0.37 (0.56) | 0.0035 | -0.36 (0.64) | 0.0425 | -0.02 (-0.41 to 0.37) | 0.926 |
| 8 months | -0.31 (0.41) | 0.0141 | -0.38 (0.72) | 0.1097 | 0.07 (-0.40 to 0.54) | 0.761 |
| 12 months | -0.26 (0.71) | 0.2040 | -0.33 (0.59) | 0.0960 | 0.07 (-0.48 to 0.62) | 0.789 |
| **Nutritional imbalance** |  |  |  |  |  |  |
| 4 months | -0.16 (0.64) | 0.2402 | -0.17 (0.55) | 0.2192 | 0.02 (-0.38 to 0.41) | 0.933 |
| 8 months | 0.03 (0.59) | 0.8581 | -0.29 (0.34) | 0.0174 | 0.32 (-0.09 to 0.73) | 0.122 |
| 12 months | 0.01 (0.55) | 0.9235 | 0.07 (0.62) | 0.7031 | -0.06 (-0.54 to 0.42) | 0.804 |
| **Impulsive eating** |  |  |  |  |  |  |
| 4 months | -0.11 (0.65) | 0.4001 | -0.05 (0.84) | 0.8031 | -0.06 (-0.54 to 0.42) | 0.798 |
| 8 months | -0.12 (0.87) | 0.6058 | 0.05 (1.03) | 0.8663 | -0.18 (-0.96 to 0.61) | 0.646 |
| 12 months | -0.48 (0.72) | 0.0266 | -0.31 (0.67) | 0.1579 | -0.17 (-0.75 to 0.41) | 0.551 |
| **HADS-anxiety** |  |  |  |  |  |  |
| 4 months | -0.23 (1.98) | 0.4269 | 0.12 (3.02) | 0.8017 | -0.35 (-1.43 to 0.73) | 0.516 |
| 8 months | -0.33 (2.58) | 0.4076 | -0.19 (3.60) | 0.7337 | -0.14 (-1.51 to 1.22) | 0.835 |
| 12 months | -0.52 (2.77) | 0.2273 | -0.74 (3.21) | 0.1655 | 0.21 (-1.12 to 1.54) | 0.751 |
| **HADS-depression** |  |  |  |  |  |  |
| 4 months | -1.42 (2.51) | 0.0003 | -1.19 (2.95) | 0.0116 | -0.23 (-1.37 to 0.91) | 0.688 |
| 8 months | -1.12 (2.86) | 0.0140 | -0.64 (4.32) | 0.3407 | -0.47 (-2.06 to 1.11) | 0.552 |
| 12 months | -1.95 (2.85) | <.0001 | -2.38 (3.39) | <.0001 | 0.43 (-0.95 to 1.81) | 0.535 |
| **PSQI** |  |  |  |  |  |  |
| 4 months | -0.43(2.44) | 0.2377 | -0.95 (2.39) | 0.0134 | 0.53 (-0.49 to 1.55) | 0.307 |
| 8 months | 0.05 (2.51) | 0.9037 | -0.73 (3.24) | 0.1560 | 0.78 (-0.48 to 2.03) | 0.221 |
| 12 months | -0.67 (2.13) | 0.0487 | -0.89 (3.62) | 0.1364 | 0.23 (-1.12 to 1.58) | 0.729 |
| **KOQOL** |  |  |  |  |  |  |
| 4 months | -5.88 (9.59) | 0.0064 | -2.19 (7.19) | 0.2421 | -3.69 (-9.38 to 2.01) | 0.198 |
| 8 months | -4.93 (7.48) | 0.0283 | -4.00 (7.24) | 0.0967 | -0.93 (-7.08 to 5.22) | 0.757 |
| 12 months | -5.14 (9.05) | 0.0533 | -4.36 (6.62) | 0.0537 | -0.78 (-7.52 to 5.96) | 0.813 |

PSVLCD, protein-supplemented very-low-calorie diet program; GPAQ, Global Physical Activity Questionnaires; HADS, Hospital Anxiety and Depression Scale; PSQI, Pittsburg Sleep Quality Index; KOQOL, Korean version of Obesity-related Quality of Life Scale

**Supplementary Table 4. Body weight and body composition with the diet intervention according to BMI group and gender**

| Variables | PSVLCD group (N = 48) | | | Control group (N = 45) | | | Difference between groups (95% CI) | p-value |  |  |
| --- | --- | --- | --- | --- | --- | --- | --- | --- | --- | --- |
|  | Baseline | 12 months | change | Baseline | 12 months | change |  |  |  |  |
| BMI less than 30 | PSVLCD group (N = 22) | | | Control group (N = 20) | | | Difference between groups (95% CI) | p-value |  |  |
|  | Baseline | 12 months | change | Baseline | 12 months | change |  |  |  |  |
| Body weight, kg | 73.46 (8.49) | 68.45 (10.17) | -5.55 (3.58) | 73.70 (9.74) | 69.15 (10.07) | -4.69 (4.87) | -0.87 (-3.60 to 1.86) | 0.528 |  |  |
| Percentage of weight loss (%) | 100 | 92.32 (5.39) | -7.68 (5.39) | 100 | 93.76  (6.32) | -6.24 (6.32) | -1.44 (-5.13 to 2.24) | 0.433 |  |  |
| Waist circumference, cm | 93.68 (5.67) | 85.10 (10.59) | -8.80 (7.51) | 91.64 (5.96) | 85.93 (6.76) | -5.43 (4.55) | -3.37 (-7.22 to 0.47) | 0.084 |  |  |
| Body fat mass, kg | 25.69 (5.33) | 22.03 (4.58) | -3.76 (3.59) | 26.36 (4.52) | 22.40 (3.45) | -3.92 (4.64) | 0.16 (-2.45 to 2.77) | 0.904 |  |  |
| Lean body mass, kg | 26.35 (5.06) | 25.64(5.48) | -1.00 (1.20) | 26.05 (5.22) | 25.68 (5.60) | -0.49 (0.85) | -0.51 (-1.16 to 0.13) | 0.113 |  |  |
| Percent body fat, % | 35.22 (5.92) | 32.40 (6.01) | -2.72 (3.77) | 35.95 (5.44) | 32.69 (4.93) | -3.15 (4.45) | 0.43 (-2.14 to 2.99) | 0.369 |  |  |
| Visceral fat area, cm^2^ | 93.25 (23.92) | 78.03 (28.46) | -17.11 (12.68) | 103.76 (25.91) | 95.03 (26.59) | -10.43 (26.18) | -6.68 (-22.54 to 9.18) | 0.197 |  |  |
| BMI more than 30 | PSVLCD group (N = 23) | | | Control group (N = 23) | | | Difference between groups (95% CI) | p-value |  |  |
|  | Baseline | 12 months | change | Baseline | 12 months | change |  |  |  |  |
| Body weight, kg | 94.37 (14.36) | 85.12 (14.05) | -8.22 (8.86) | 94.72 (15.99) | 90.35 (16.73) | -4.63 (6.17) | -3.59 (-8.36 to 1.16) | 0.134 |  |  |
| Percentage of weight loss (%) | 100 | 91.23 (8.66) | -8.76 (8.66) | 100 | 95.26  (6.51) | -4.74 (6.51) | -4.02 (-8.80 to 0.76) | 0.087 |  |  |
| Waist circumference, cm | 107.13(7.56) | 98.75 (9.26) | -7.87 (7.02) | 107.17 (7.94) | 102.67 (9.71) | -4.30 (8.06) | -3.56 (-8.28 to 1.16) | 0.135 |  |  |
| Body fat mass, kg | 38.50 (8.17) | 30.90 (8.65) | -7.10 (7.20) | 39.39 (8.66) | 35.42 (9.61) | -3.98 (7.15) | -3.12 (-7.59 to 1.35) | 0.165 |  |  |
| Lean body mass, kg | 31.25 (7.10) | 30.15 (7.10) | -0.78 (1.58) | 32.53 (9.69) | 30.52 (6.32) | -2.35 (10.23) | 1.57 (-3.13 to 6.26) | 0.495 |  |  |
| Percent body fat, % | 40.97 (6.88) | 36.31 (7.85) | -4.56 (4.36) | 41.60 (4.01) | 39.12 (6.12) | -2.38 (5.52) | -2.19 (-5.29 to 0.92) | 0.081 |  |  |
| Visceral fat area, cm^2^ | 146.28 (42.22) | 107.32 (27.75) | -38.71 (40.84) | 148.79 (48.32) | 130.35 (41.89) | -16.08 (20.06) | -22.63 (-46.69 to 1.44) | 0.064 |  |  |
| Female | | PSVLCD group (N = 32) | | | Control group (N = 32) | | | Difference between groups (95% CI) | p-value |  |
|  | | Baseline | 12 months | change | Baseline | 12 months | change |  |  |  |
| Body weight, kg | | 78.33 (13.30) | 71.47 (13.29) | -7.75 (7.69) | 78.46 (12.30) | 73.64 (12.81) | -4.87 (5.76) | -2.88 (-6.48 to 0.71) | 0.069 |  |
| Percentage of weight loss (%) | | 100 | 90.45 (7.80) | -9.55 (7.80) | 100 | 93.89 (6.89) | -6.11 (6.89) | -3.44 (-7.34 to 0.46) | 0.0827 |  |
| Waist circumference, cm | | 97.97 (9.68) | 88.89 (13.08) | -9.61 (8.11) | 96.53 (8.96) | 91.39 (10.25) | -4.93 (6.52) | -4.67 (-8.58 to -0.78) | 0.0196 |  |
| Body fat mass, kg | | 32.60 (8.89) | 27.45 (8.86) | -5.88 (6.35) | 31.82 (8.18) | 28.22 (8.41) | -3.67 (6.22) | -2.21 (-5.55 to 1.12) | 0.189 |  |
| Lean body mass, kg | | 25.05 (3.62) | 24.00 (3.61) | -1.15 (1.21) | 26.82 (8.27) | 24.83 (3.43) | -2.12 (8.61) | -0.97 (-2.32 to 4.26) | 0.558 |  |
| Percent body fat, % | | 41.10 (5.06) | 37.72 (5.85) | -3.82 (4.04) | 40.20 (4.47) | 37.70 (5.43) | -2.56 (5.36) | -1.27 (-3.79 to 1.26) | 0.319 |  |
| Visceral fat area, cm^2^ | | 112.15 (42.52) | 87.15 (30.15) | -28.74 (35.66) | 116.92 (39.25) | 103.93 (36.27) | -11.62 (24.60) | -17.12 (-36.39 to 2.15) | 0.080 |  |
| Male | | | PSVLCD group (N = 16) | | | Control group (N = 13) | | | Difference between groups (95% CI) | p-value |
|  | | | Baseline | 12 months | change | Baseline | 12 months | change |  |  |
| Body weight, kg | | | 93.78 (15.22) | 86.15 (12.63) | -5.19 (4.62) | 99.18 (18.09) | 95.39 (17.90) | -4.14 (5.04) | -1.04 (-4.88 to 2.79) | 0.579 |
| Percentage of weight loss (%) | | | 100 | 94.29 (4.91) | -5.71 (4.91) | 100 | 96.06 (4.89) | -3.94 (4.89) | -1.78 (-5.69 to 2.13) | 0.358 |
| Waist circumference, cm | | | 104.44 (7.52) | 97.13 (7.52) | -6.01 (4.44) | 107.08 (10.52) | 102.04 (12.50) | -4.71 (6.91) | -1.30 (-5.81 to 3.17) | 0.551 |
| Body fat mass, kg | | | 30.28 (9.50) | 24.32 (6.35) | -4.49 (4.78) | 35.95 (12.09) | 31.11 (12.64) | -4.64 (5.57) | 0.15 (-3.95 to 4.25) | 0.941 |
| Lean body mass, kg | | | 35.99 (4.67) | 35.01 (4.80) | -0.42 (1.60) | 35.62 (4.78) | 36.19 (4.36) | 0.19 (1.44) | -0.61 (-1.84 to 0.61) | 0.313 |
| Percent body fat, % | | | 31.73 (6.05) | 27.93 (4.56) | -3.25 (4.39) | 35.48 (6.52) | 31.83 (6.84) | -3.23 (4.11) | -0.01(-3.42 to 3.39) | 0.994 |
| Visceral fat area, cm^2^ | | | 138.03 (41.18) | 106.59 (31.43) | -27.27 (24.10) | 150.19 (50.33) | 133.12 (38.97) | -17.07 (19.93) | -10.20 (-32.29 to 11.89) | 0.342 |

All such values are presented as mean ± SE

Linear mixed-effect model adjusted to analyze the difference within each group

PSVLCD, protein-supplemented very-low-calorie diet program
